# Supplementary figures and images for: Coordinated Tbx3/Tbx5 transcriptional control of the adult ventricular conduction system
Source: eLife. 2025 Jul 24;13:RP102027. doi: 10.7554/eLife.102027 (PMC12289313; doi:10.7554/eLife.102027)

*Tbx5* locus (5' arm + Lox2272 insert)

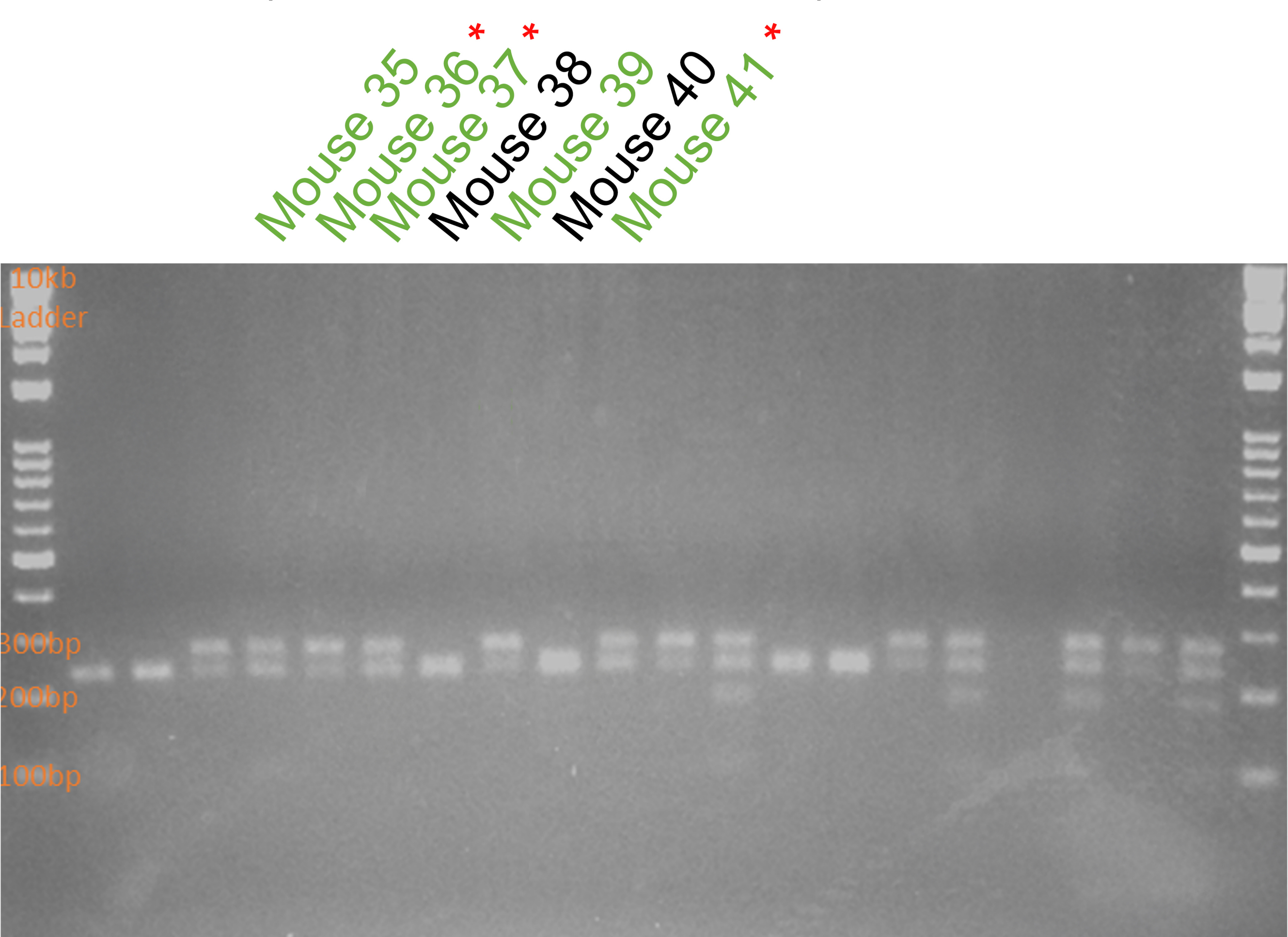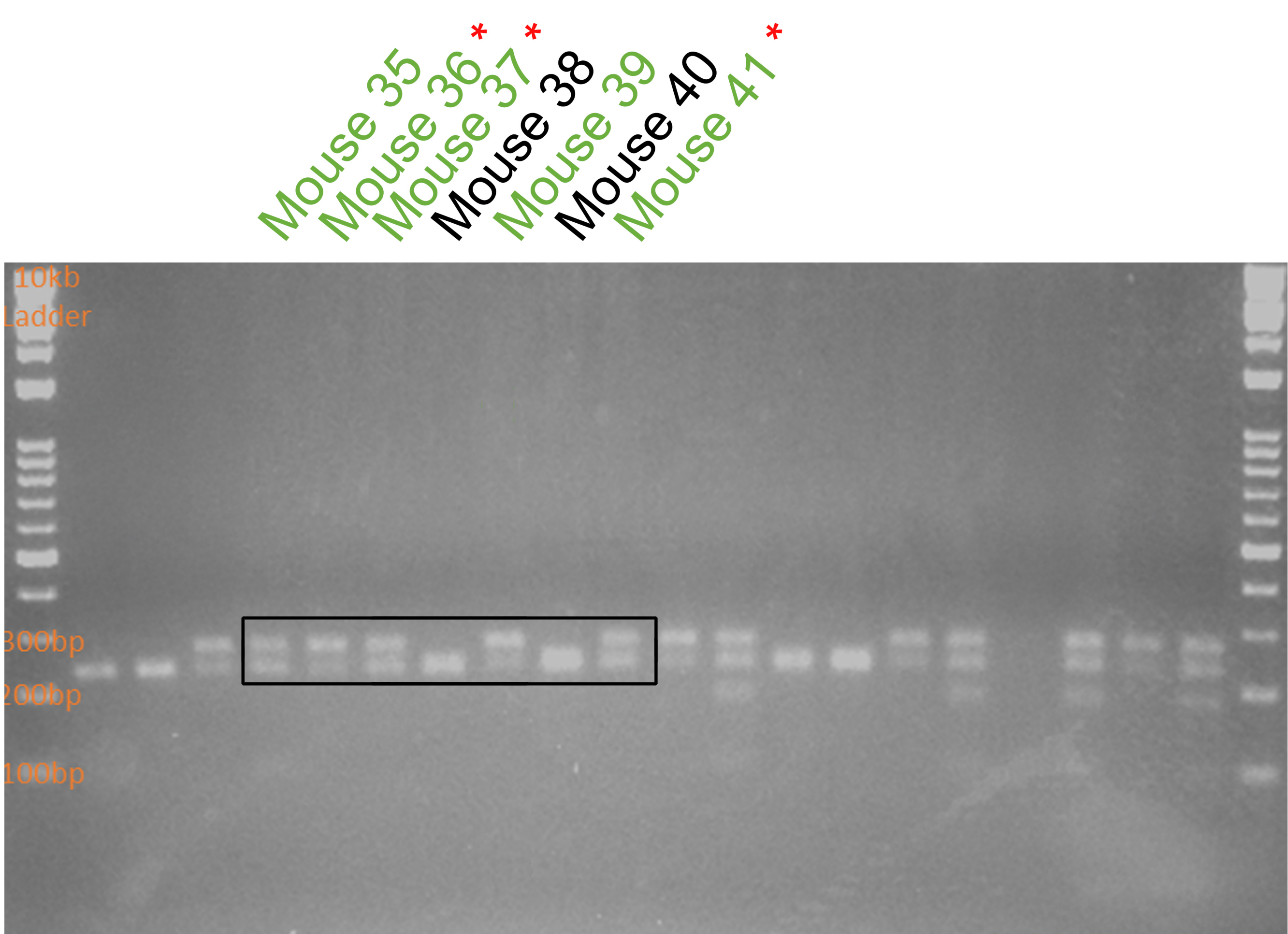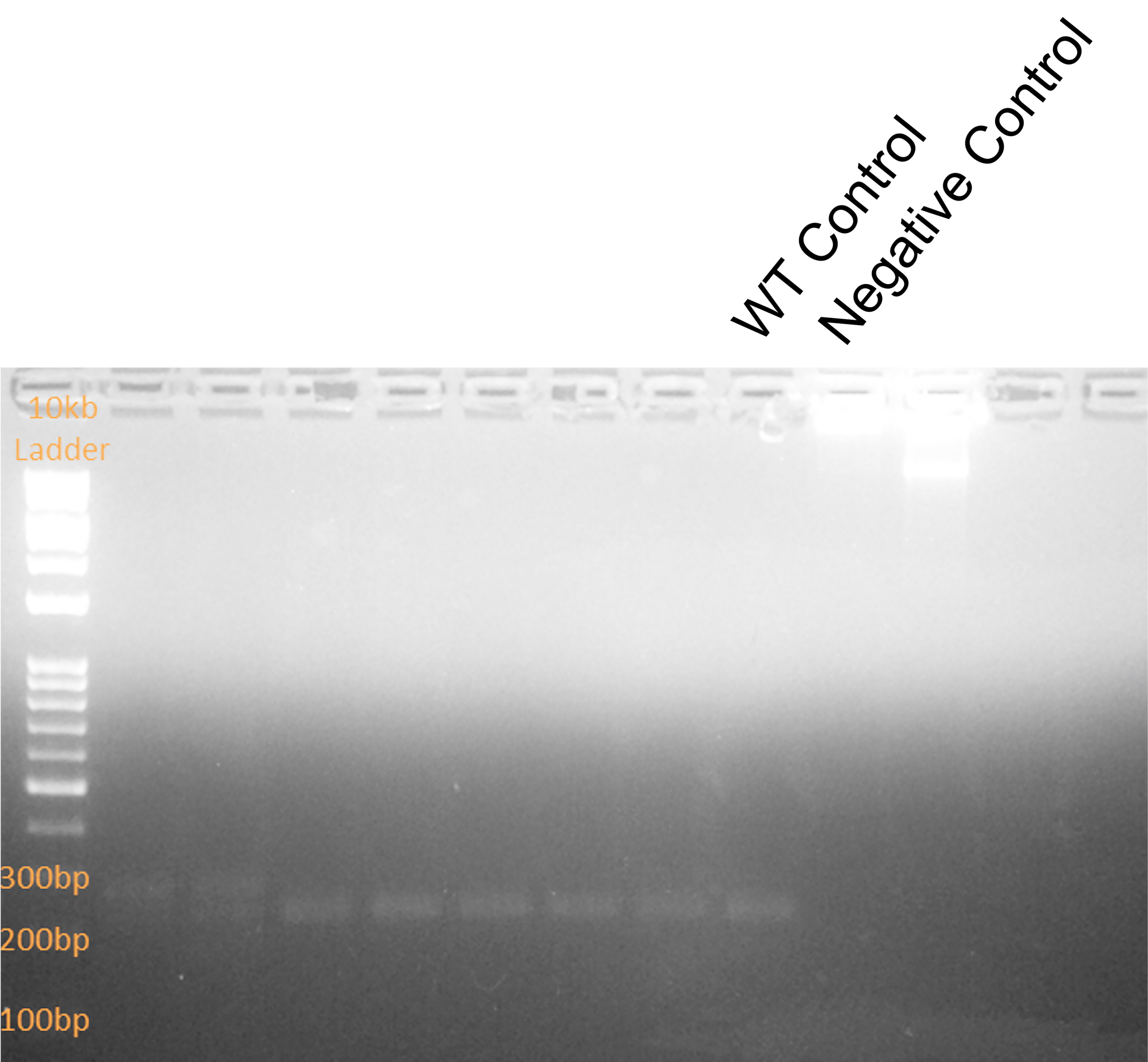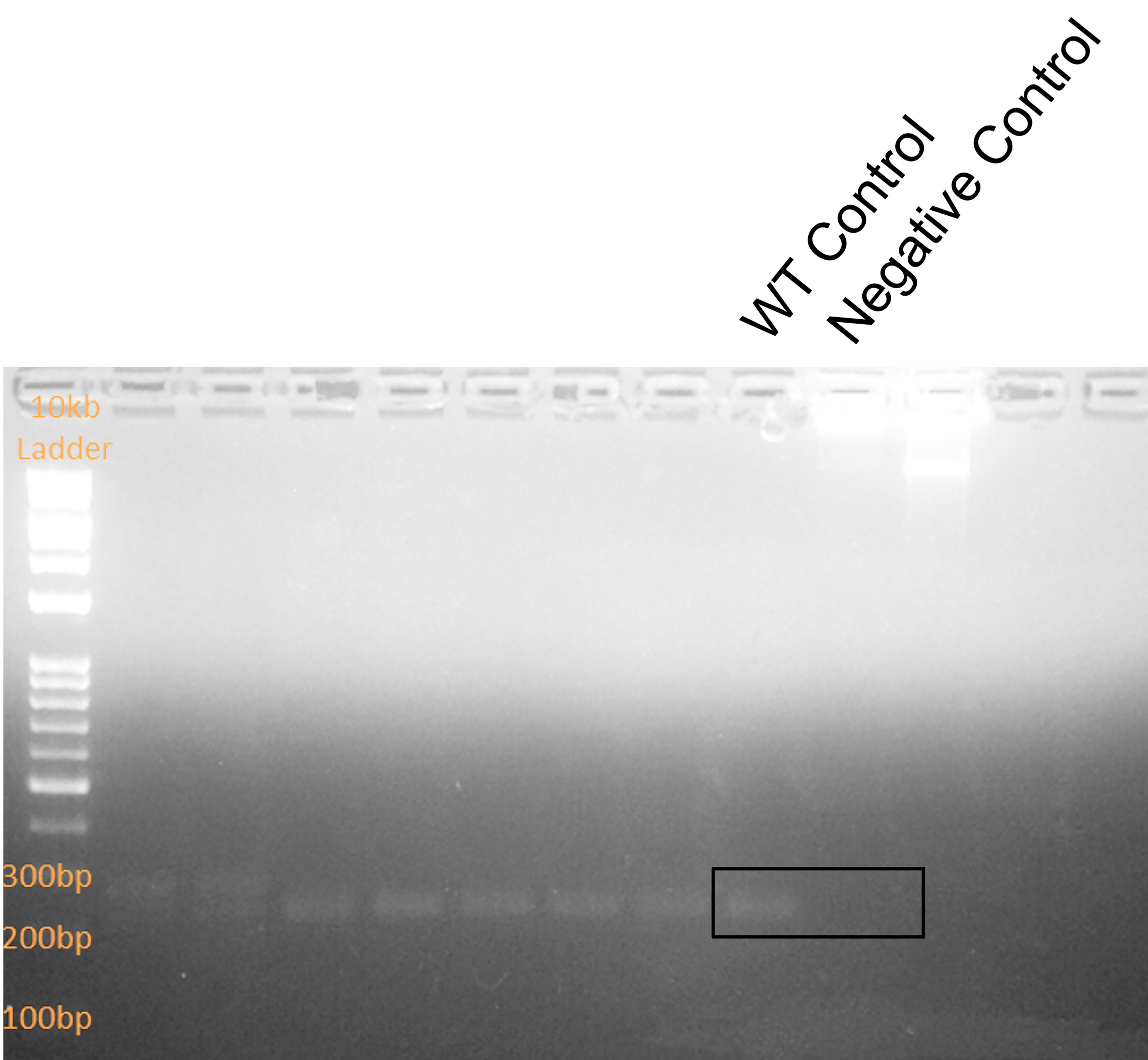

*Tbx5* locus (3' arm + Lox2272 insert)

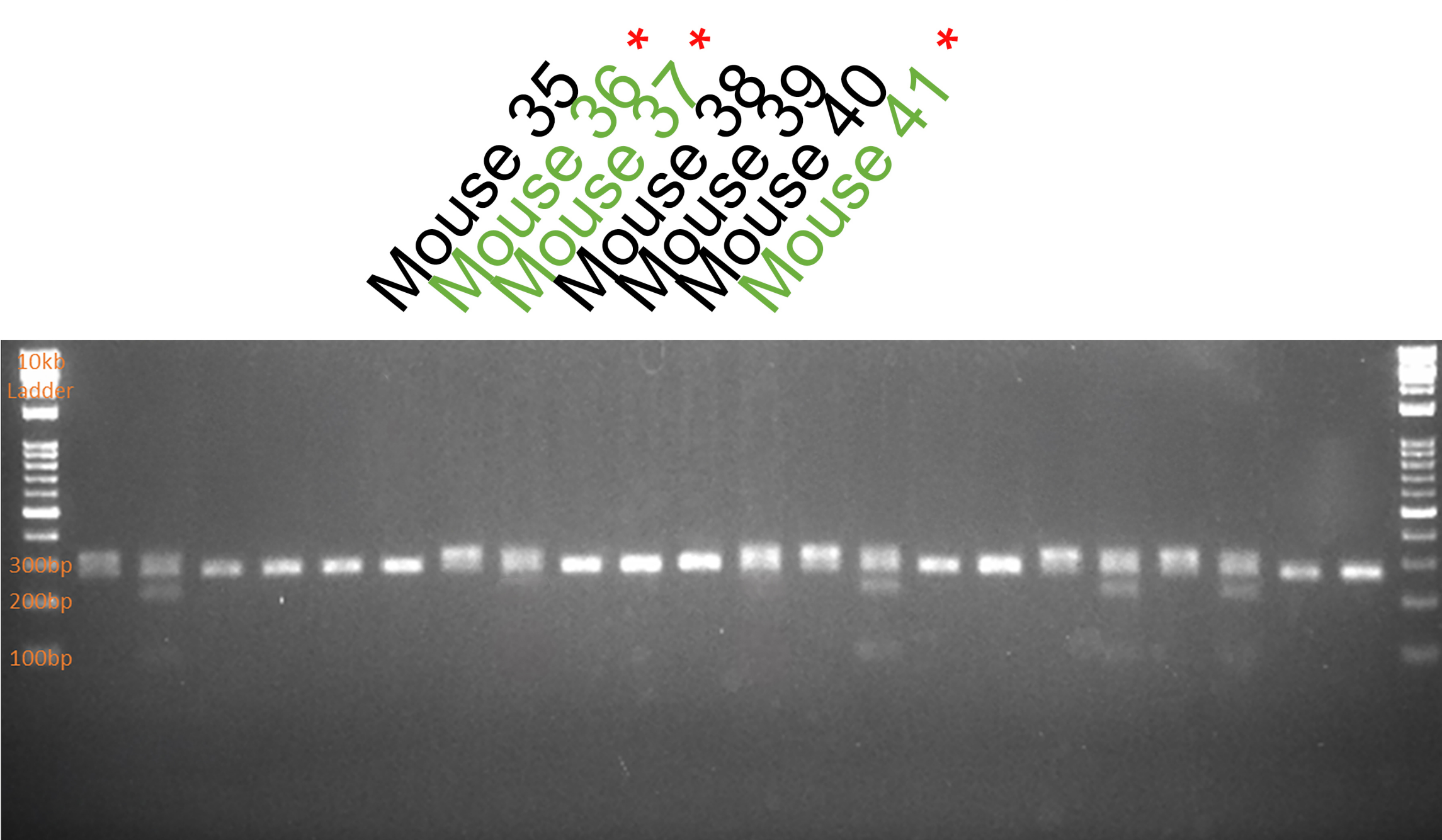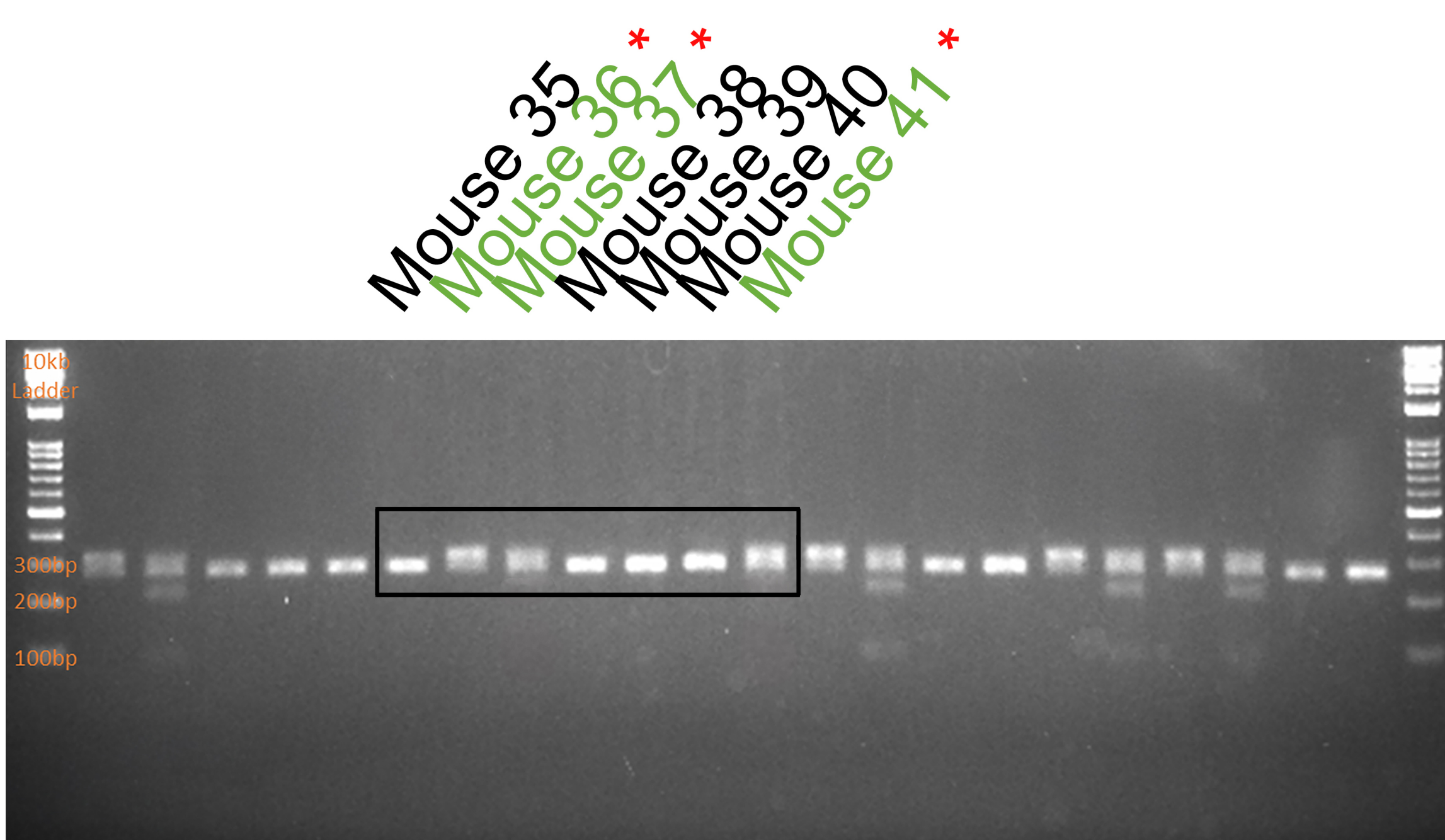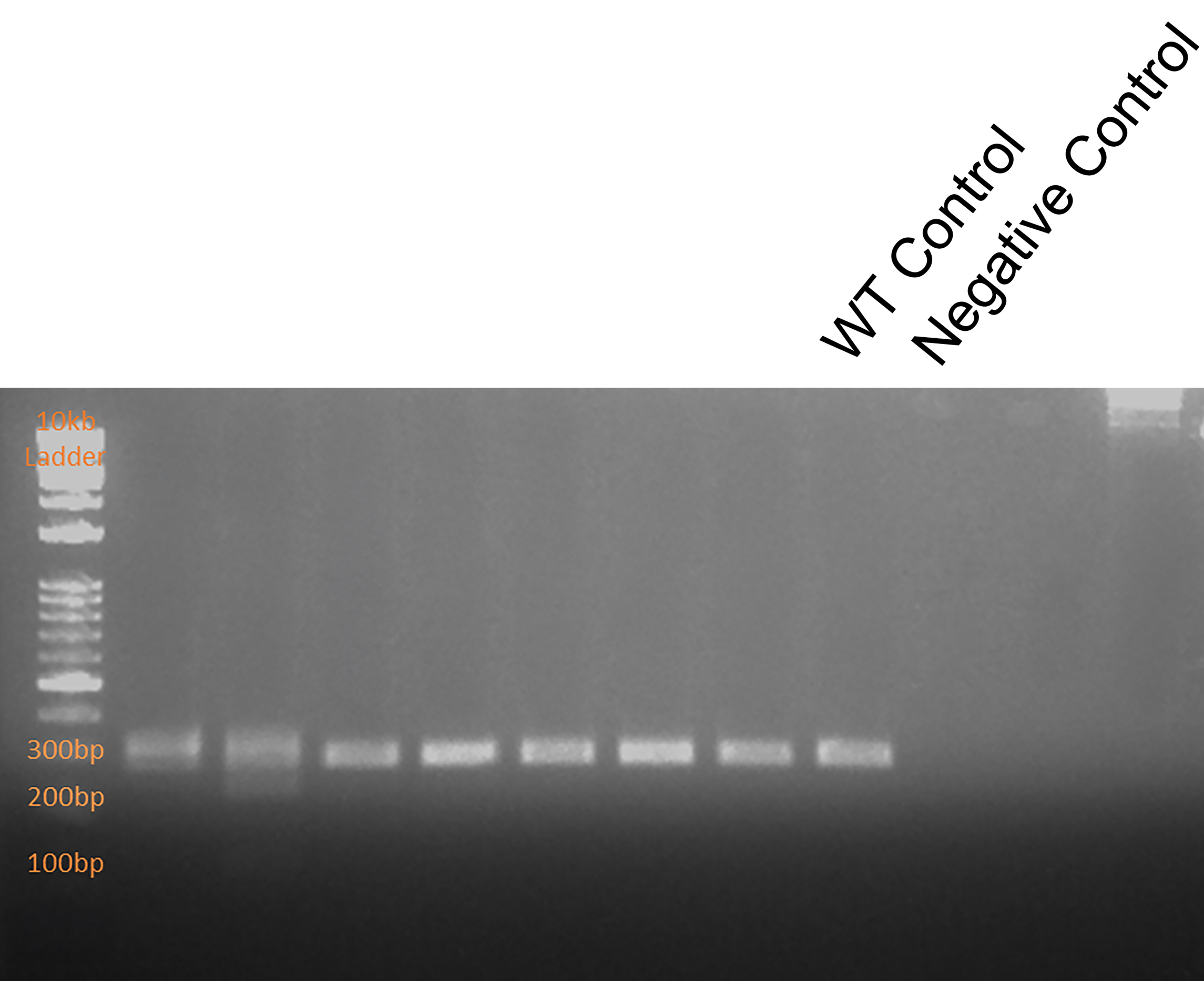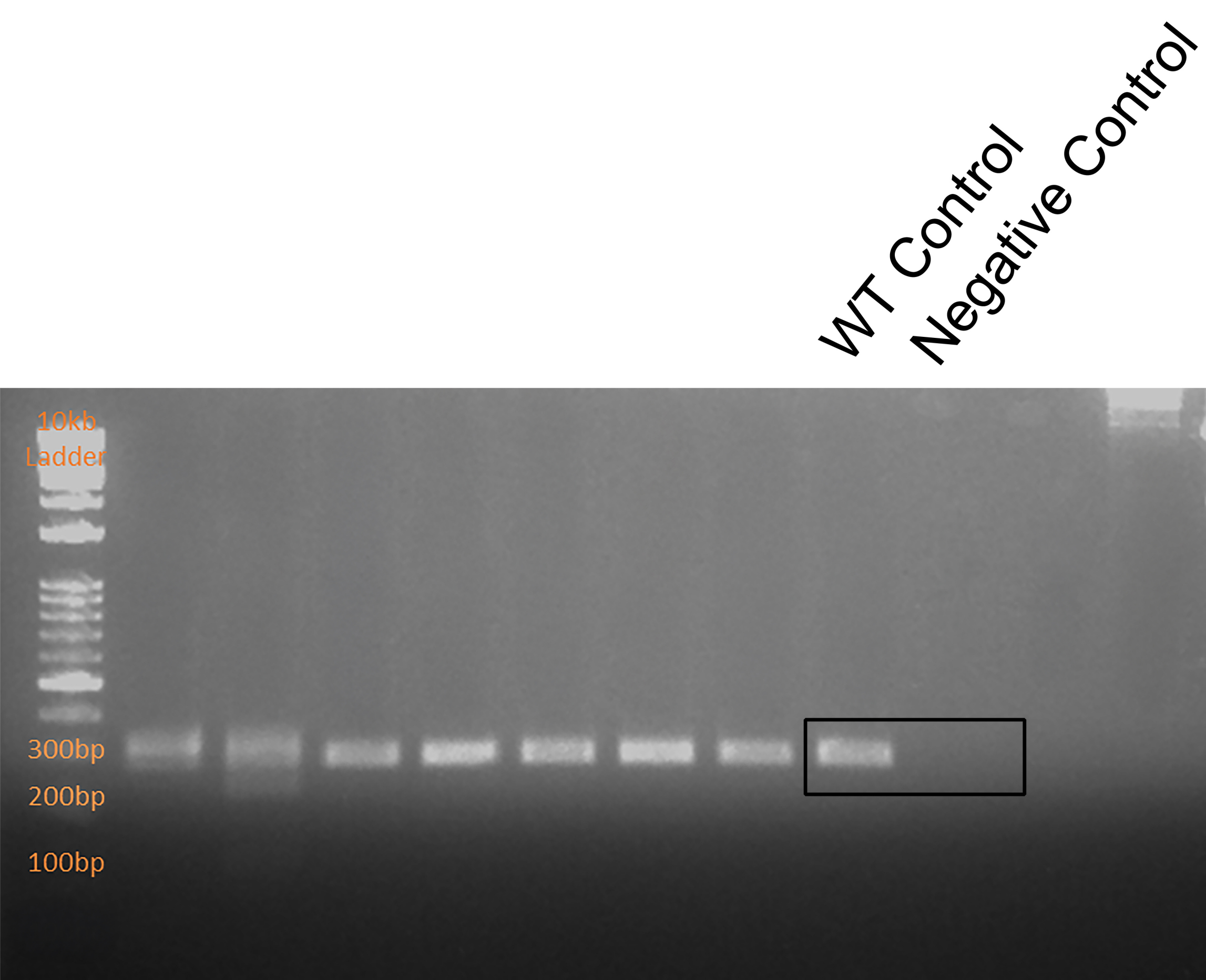

*Tbx3* locus (Frank et al., 2012)

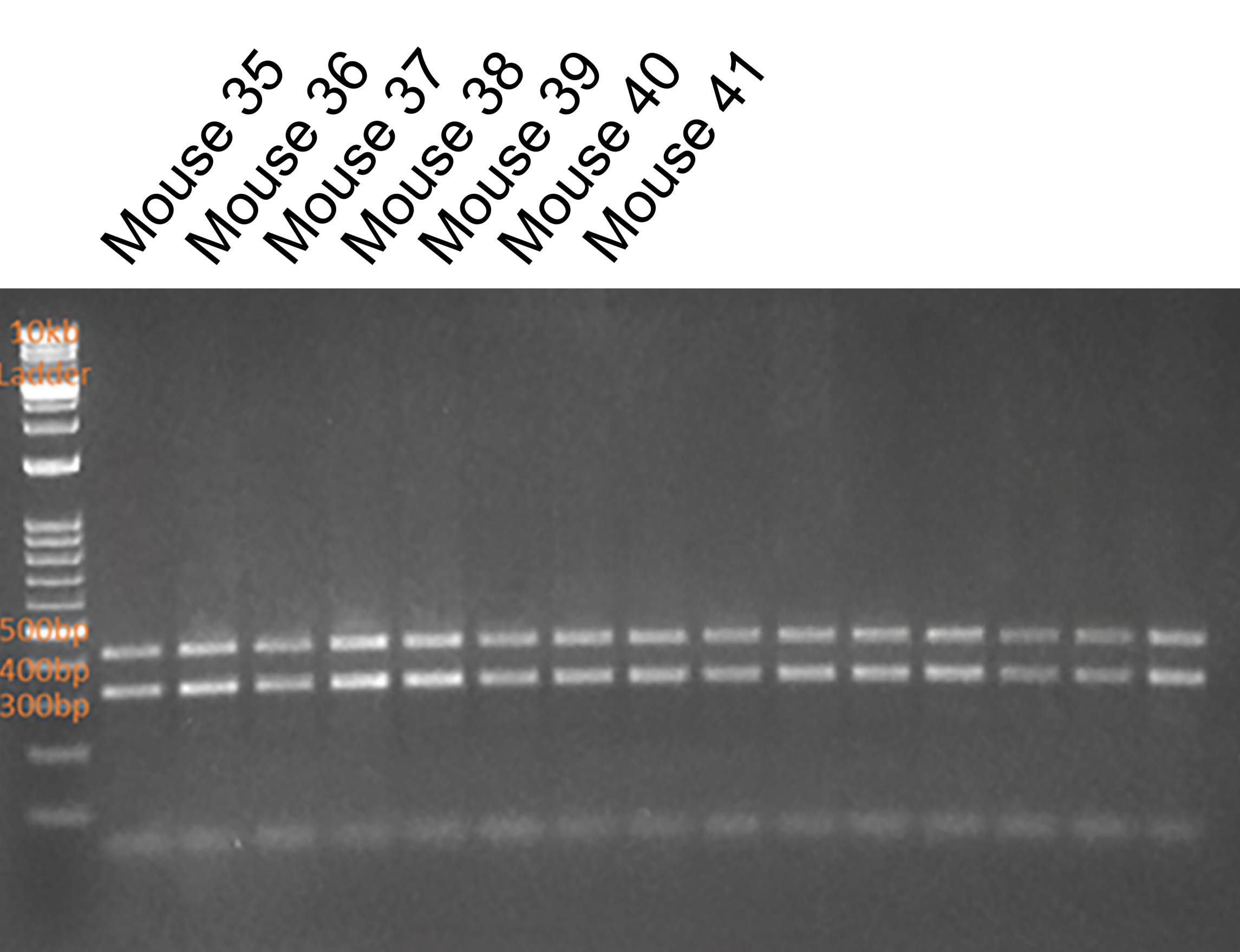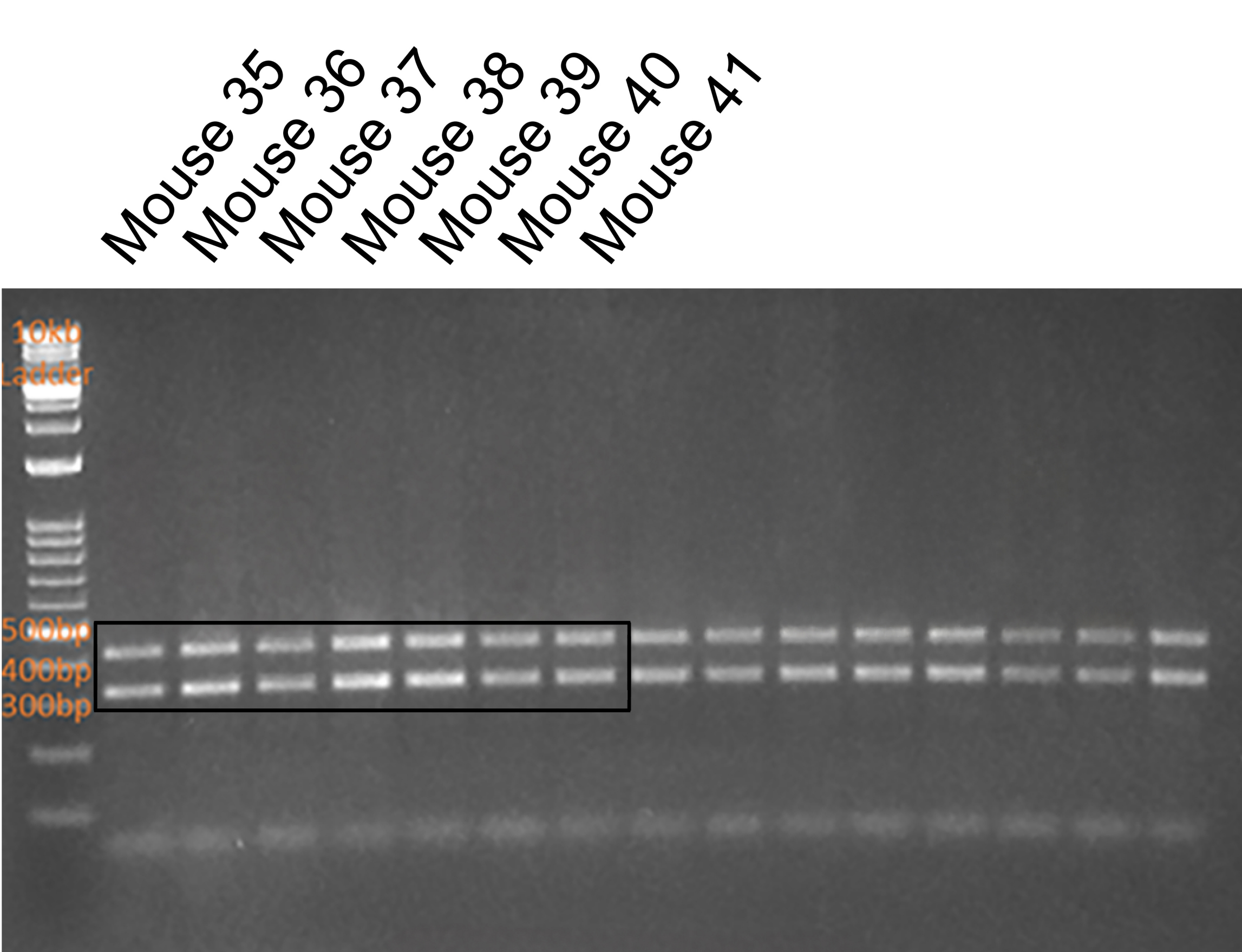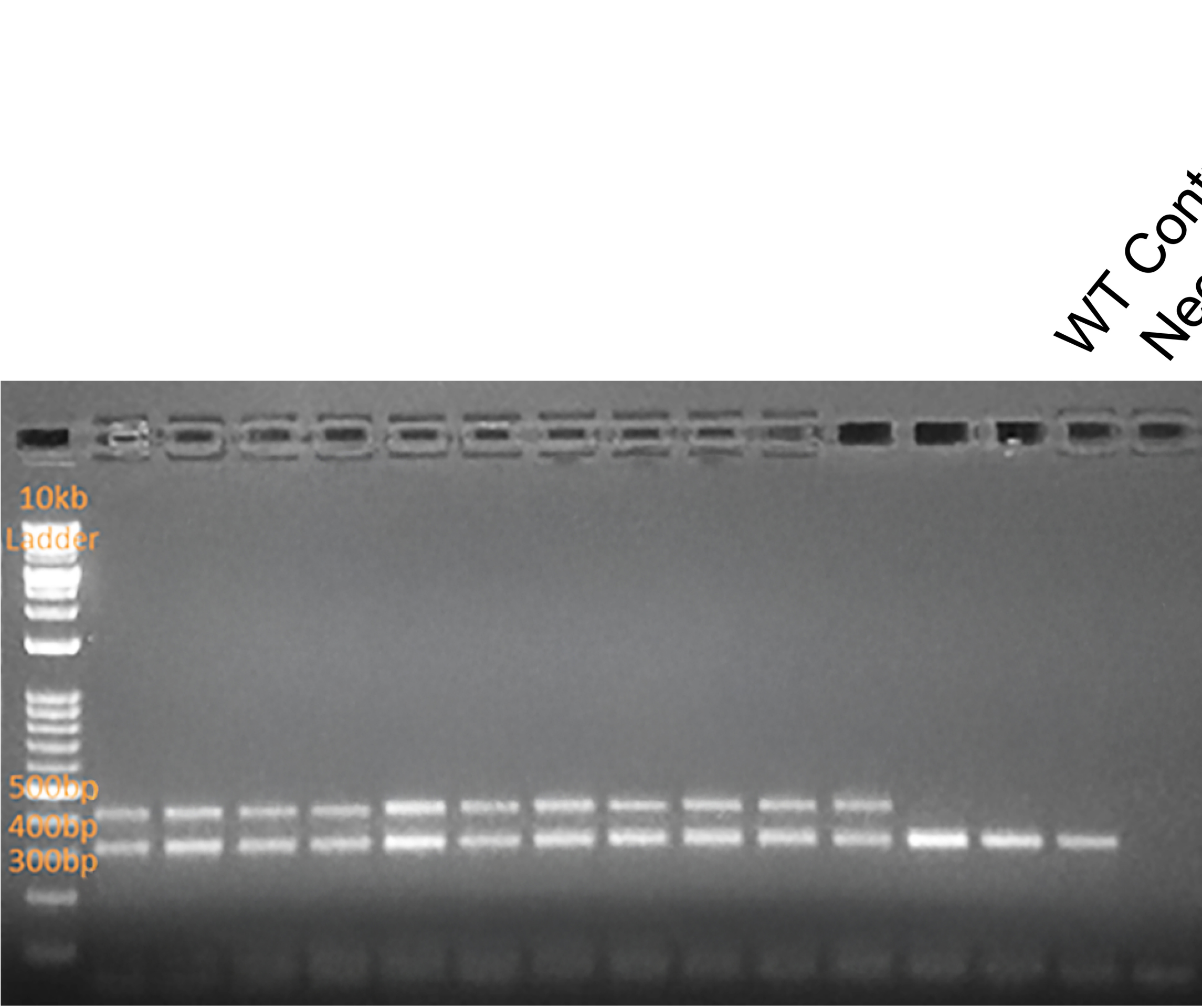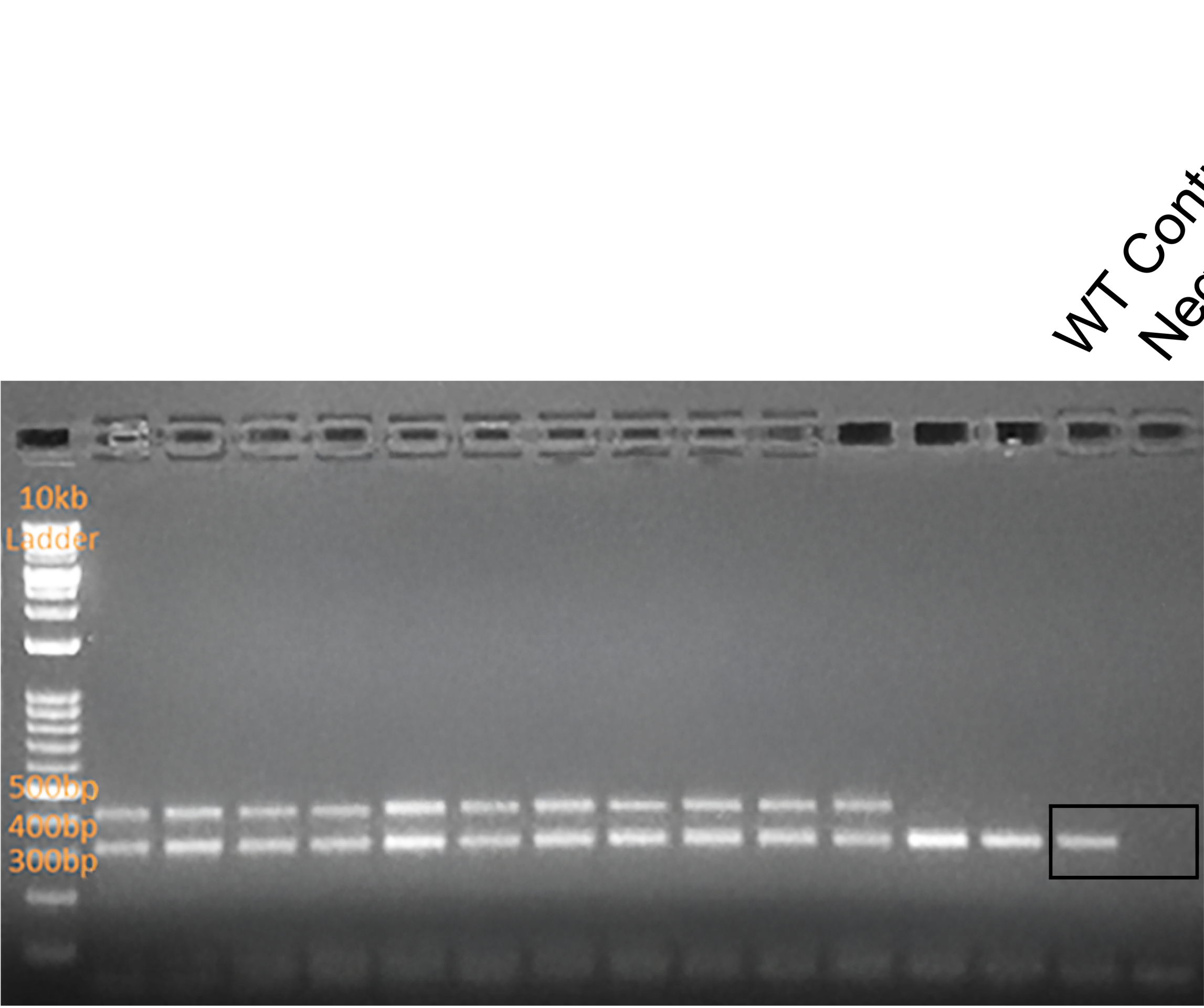

Supplement: Figure 1—figure supplement 1—source data 1. — The black rectangle indicates the region included in the final figure panel. [file elife-102027-fig1-figsupp1-data1.pdf]

*Tbx5* locus (5' arm + Lox2272 insert - cut with *EcoRI*)

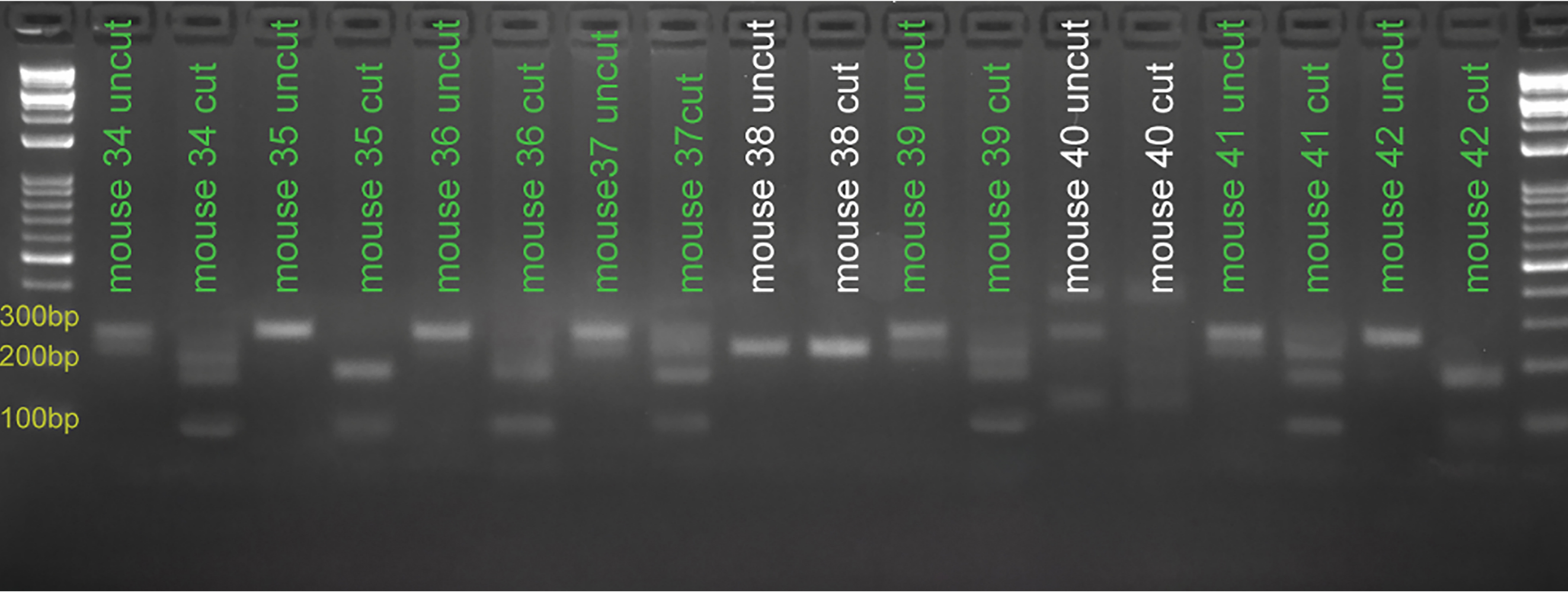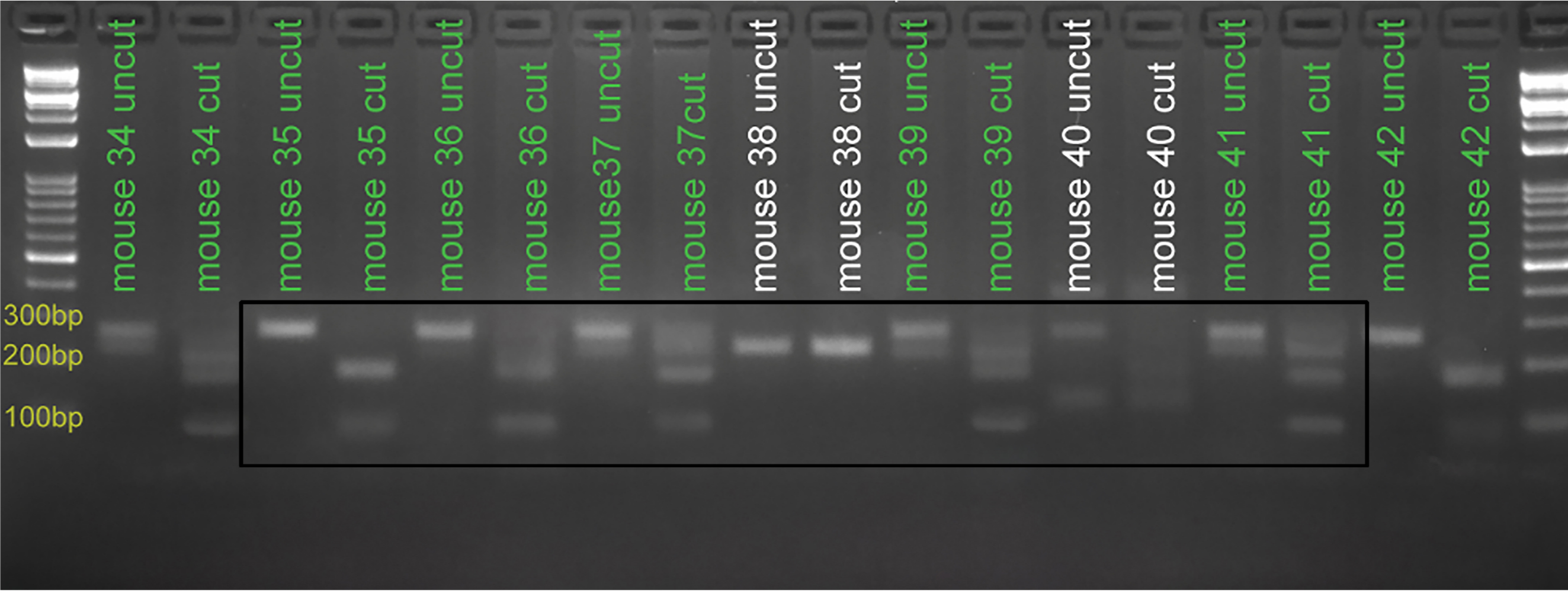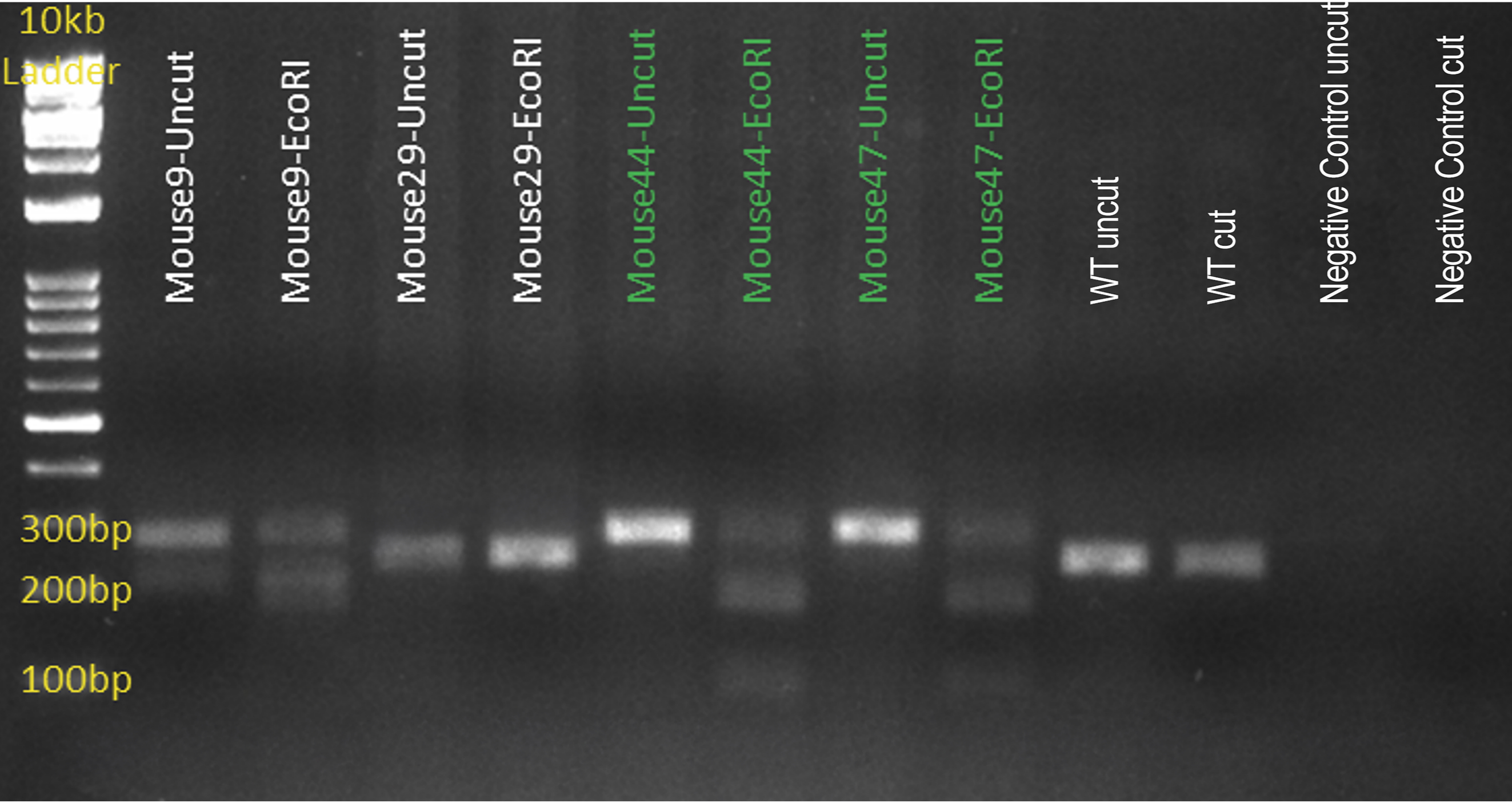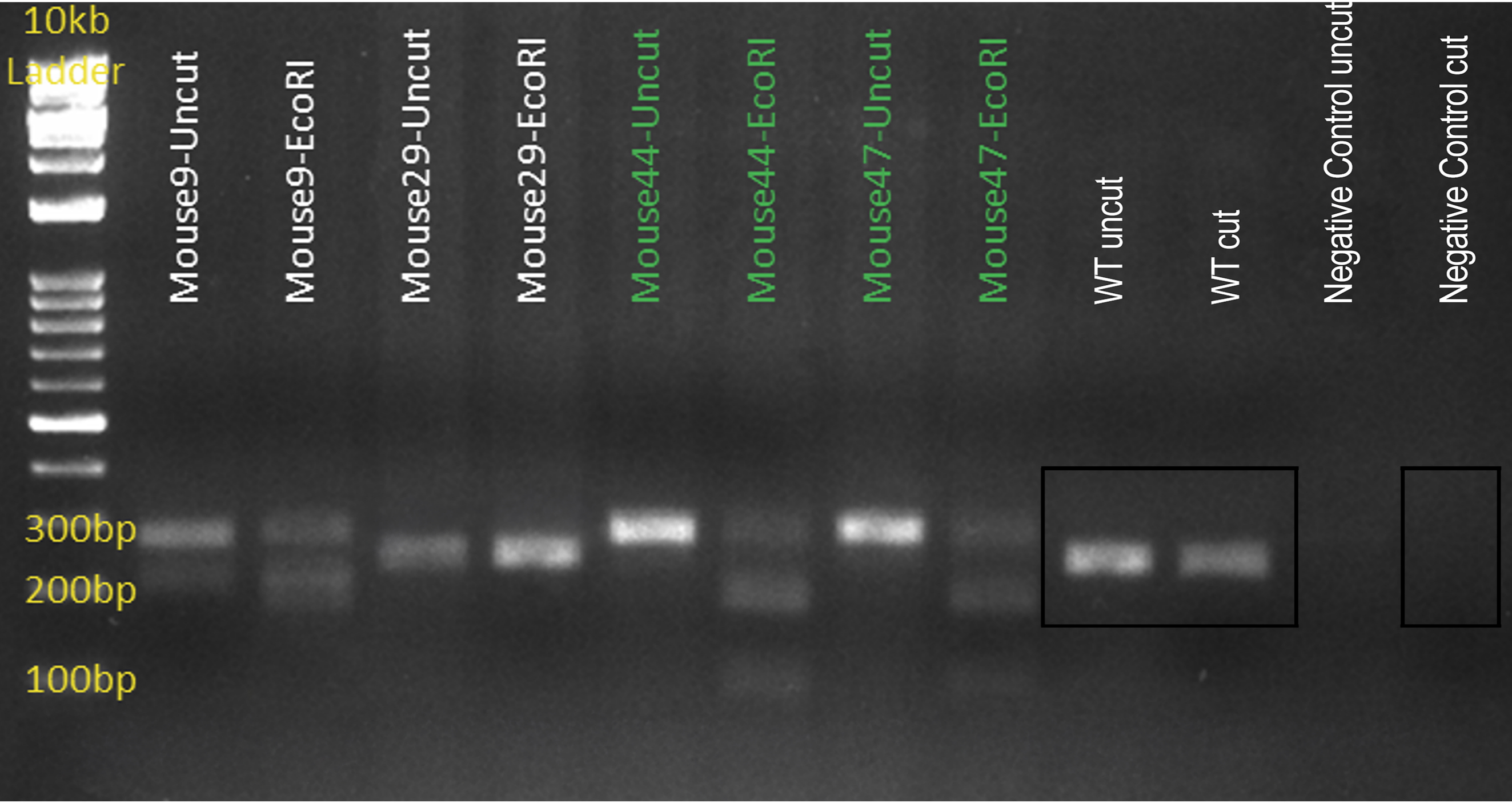

*Tbx5* locus (3' arm + Lox2272 insert - cut with *SphI*)

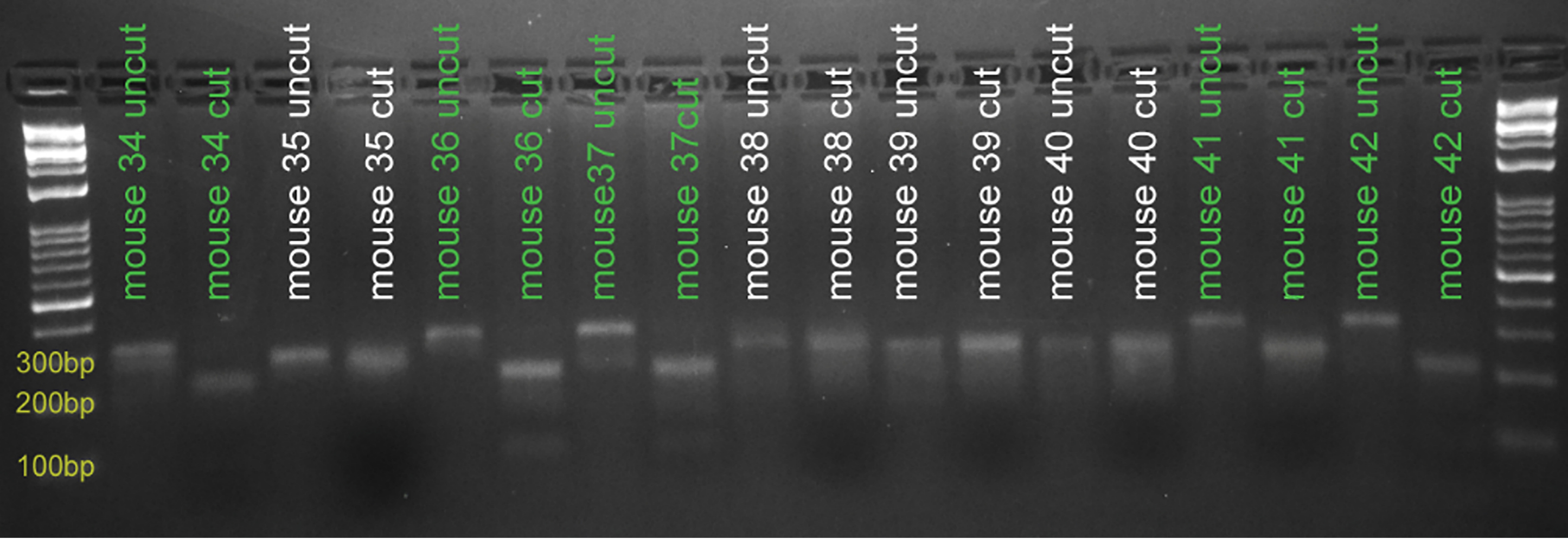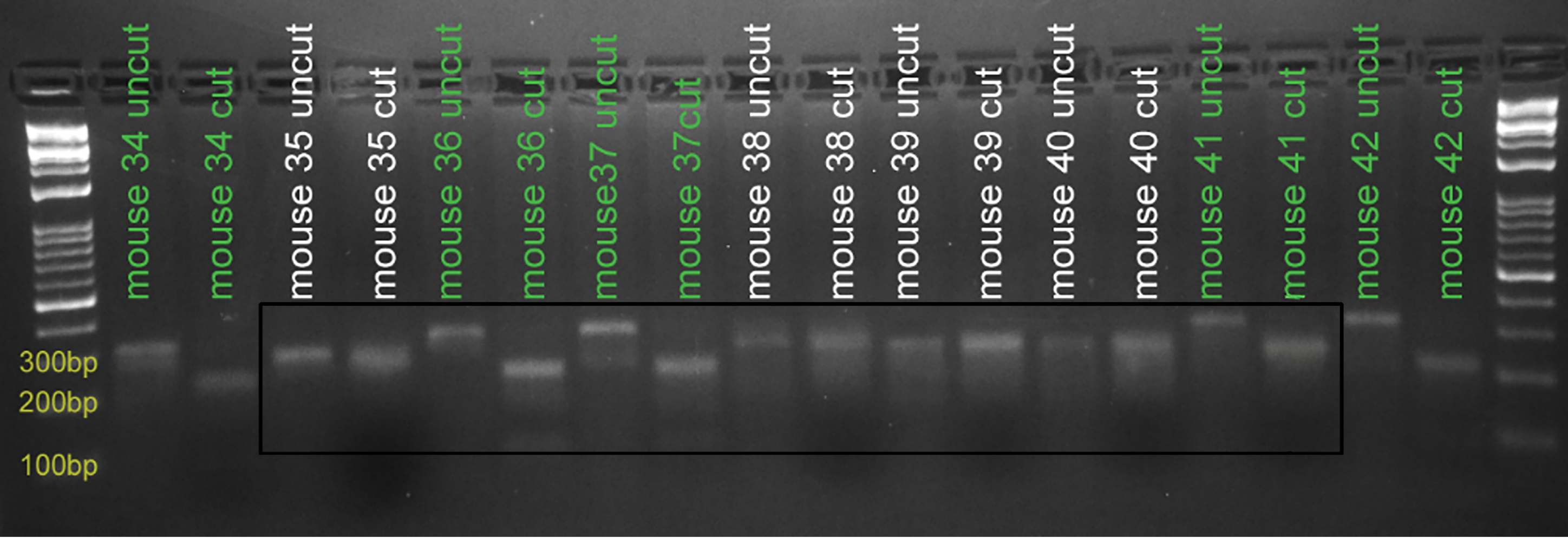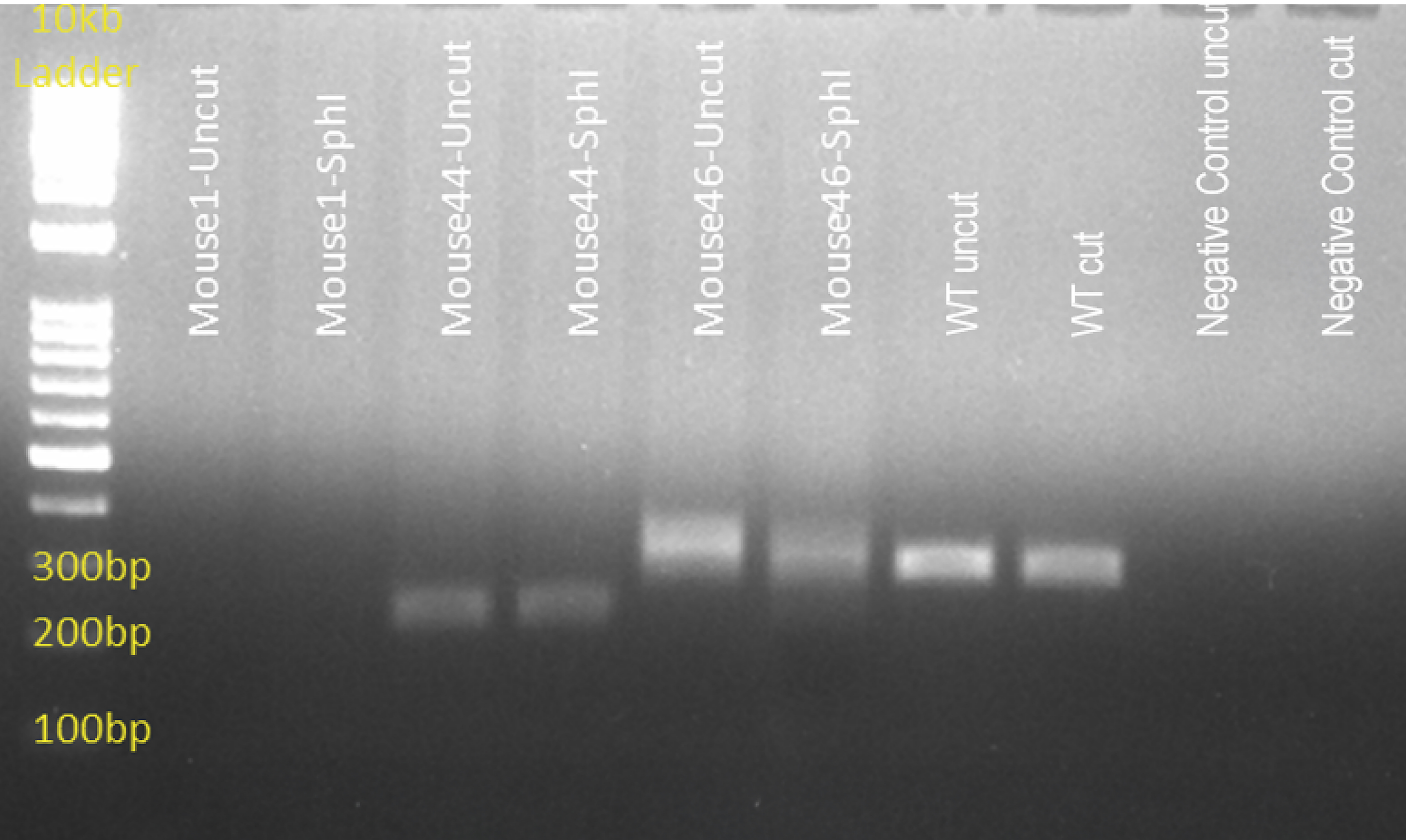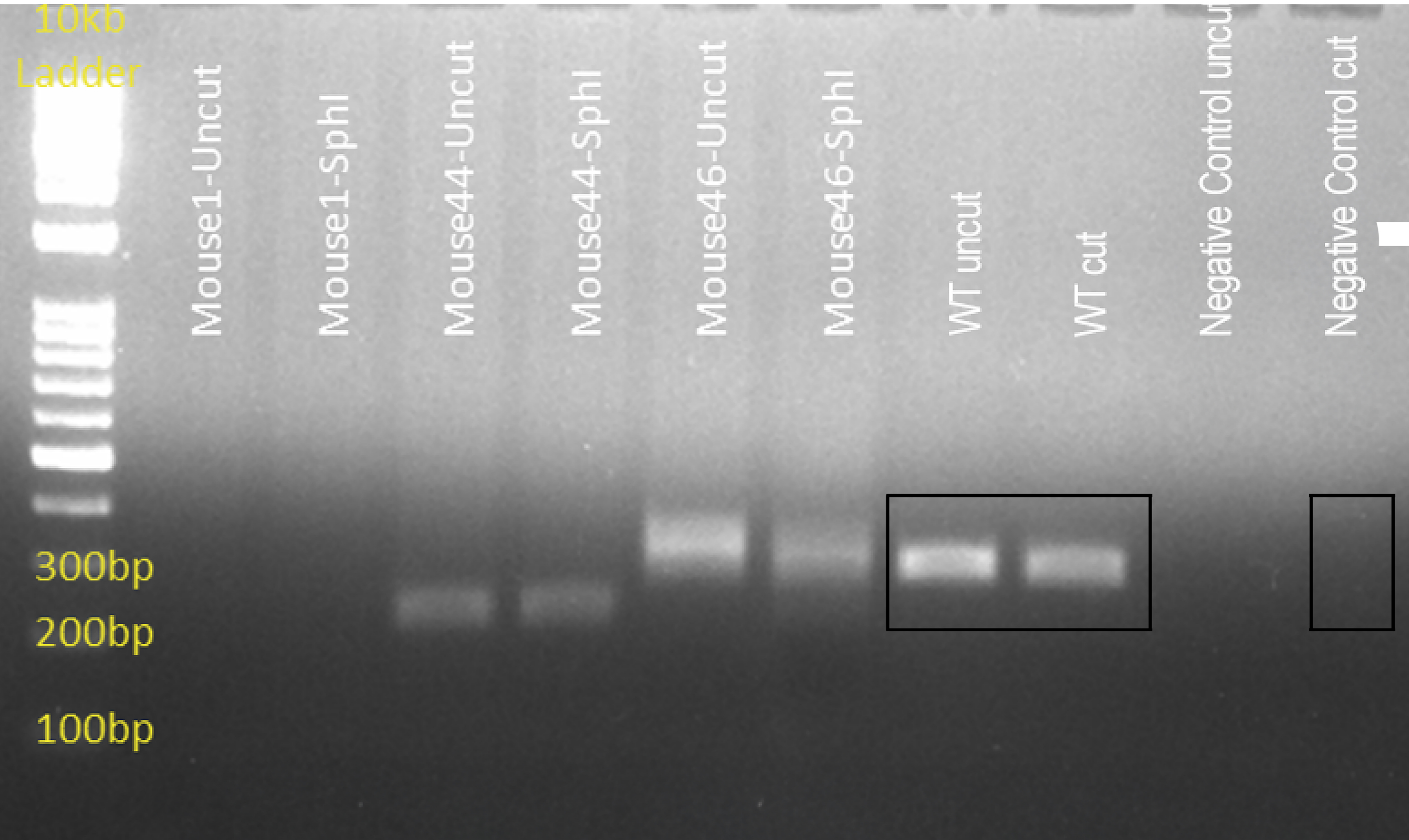

Supplement: Figure 1—figure supplement 1—source data 2. [file elife-102027-fig1-figsupp1-data2.pdf]

# Embryos: Genotyping for *Tbx5* locus

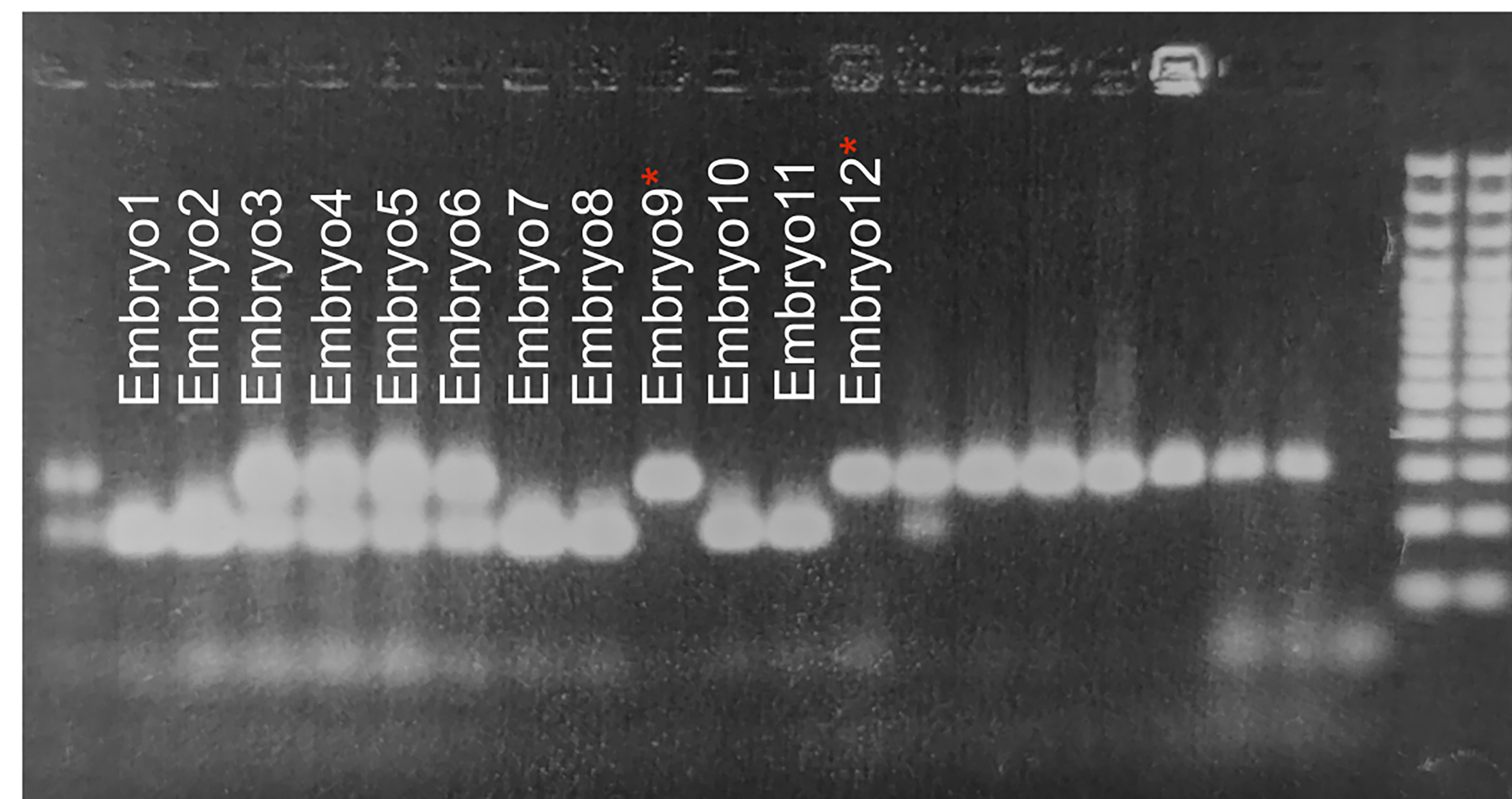

$\Delta$ Flox (Null)  
WT Control

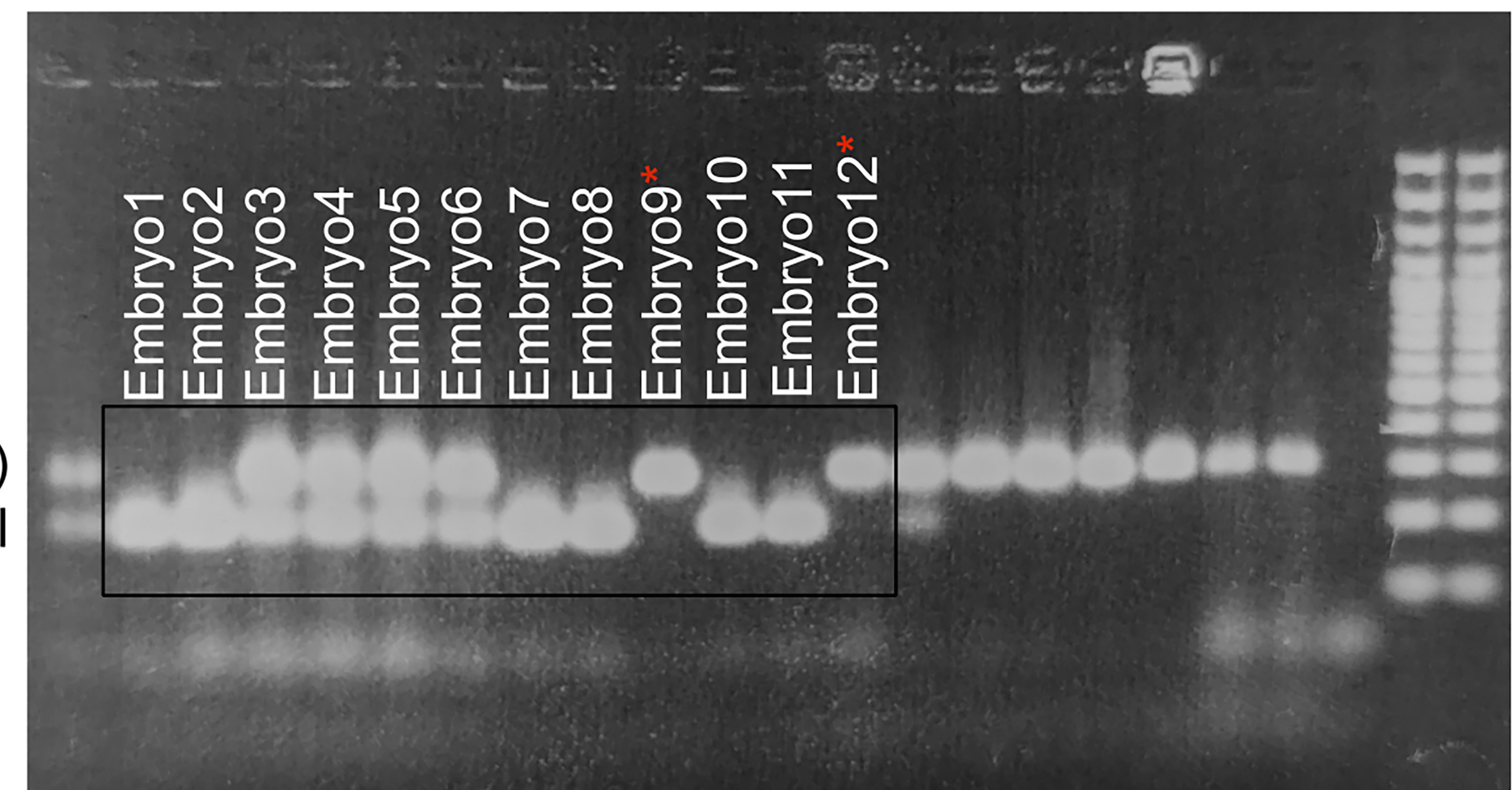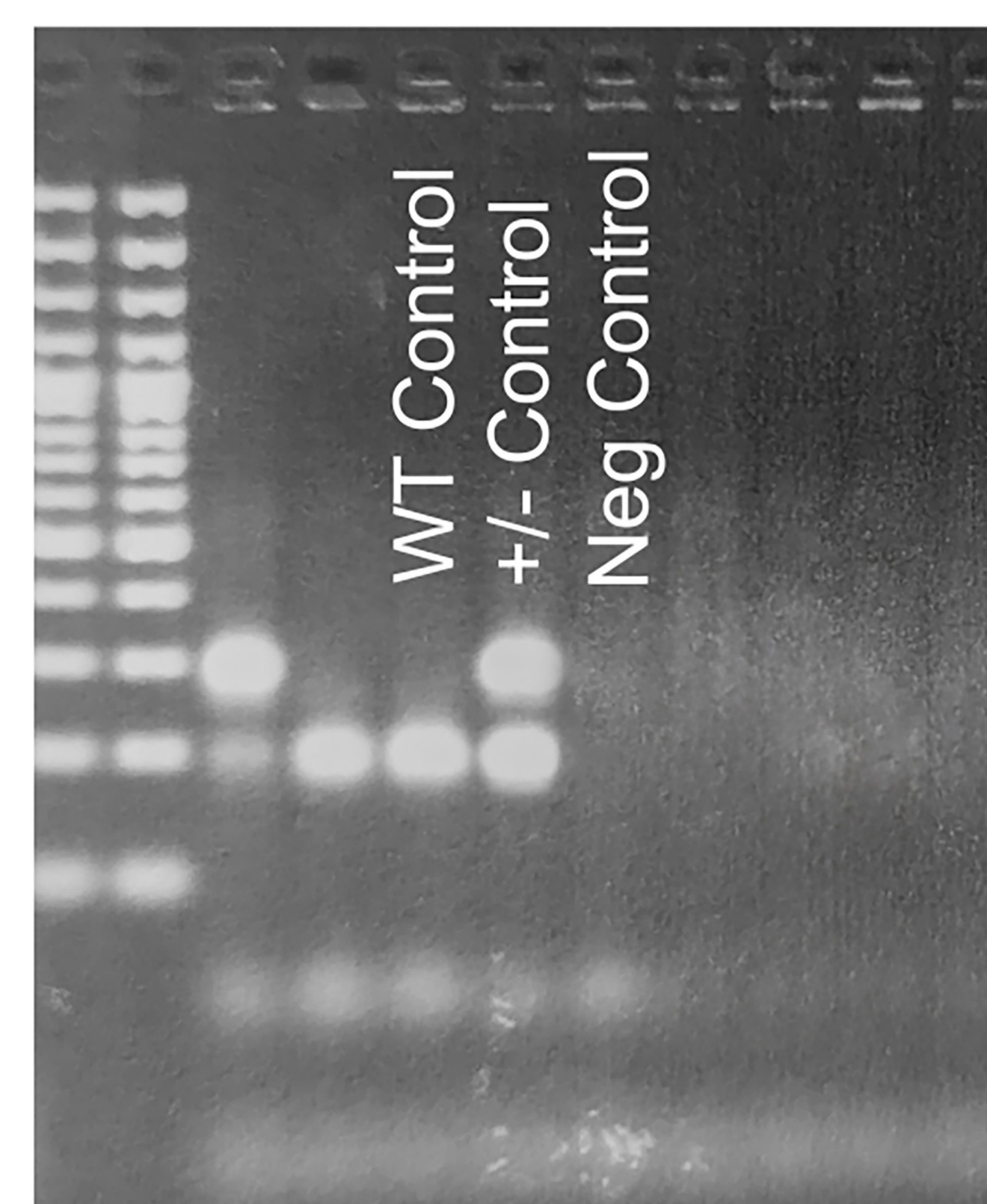

$\Delta$ Flox (Null)  
WT Control

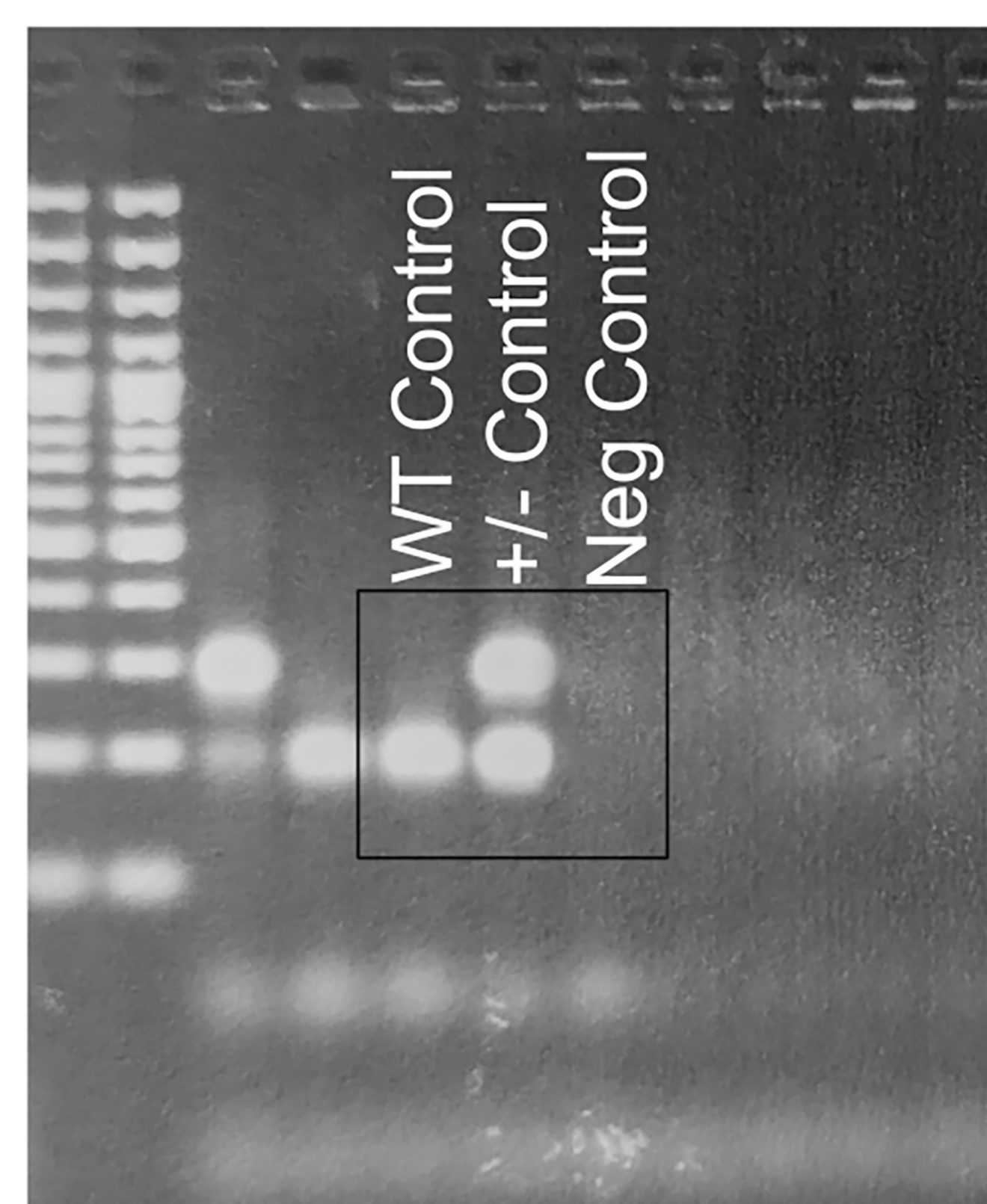

Supplement: Figure 1—figure supplement 2—source data 2. [file elife-102027-fig1-figsupp2-data2.pdf]

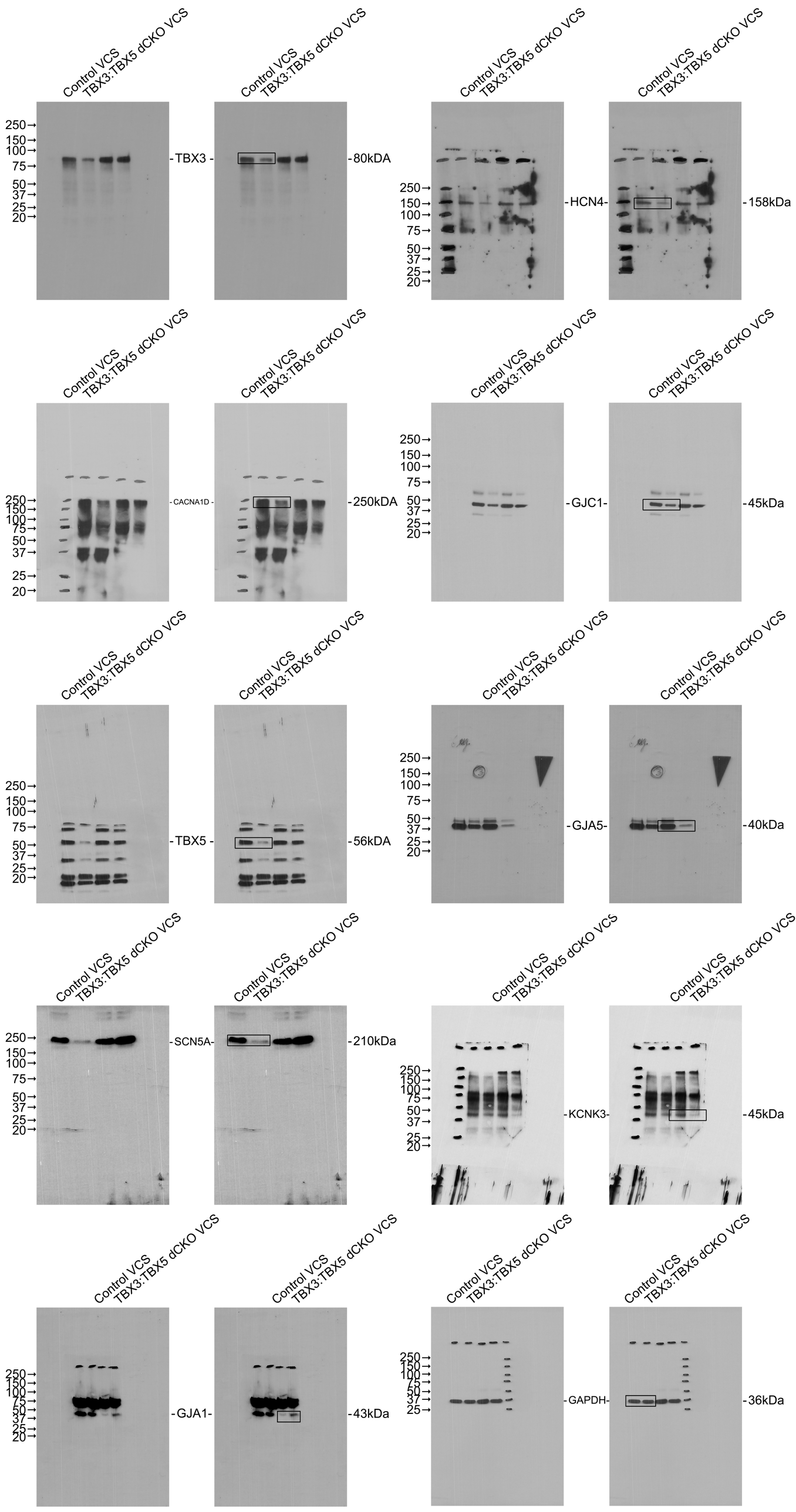

Supplement: Figure 4—source data 1. — The black rectangle indicates the region included in the final figure panel. [file elife-102027-fig4-data1.pdf]

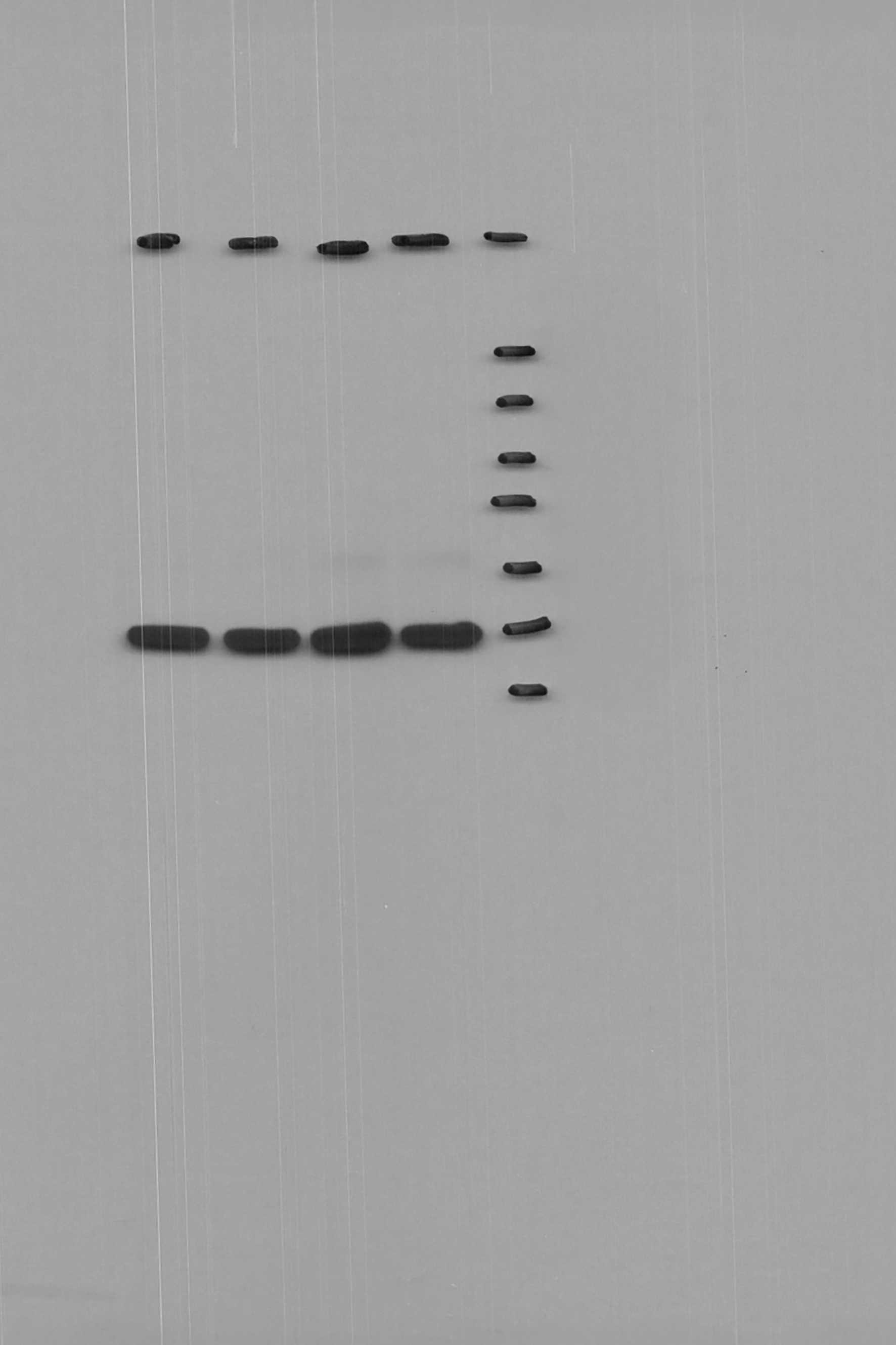

Supplement: Figure 4—source data 2. [file elife-102027-fig4-data2.zip › Figure 4-source data 2/10_GAPDH_2023_05_03_07_37_09-1_2.jpg]

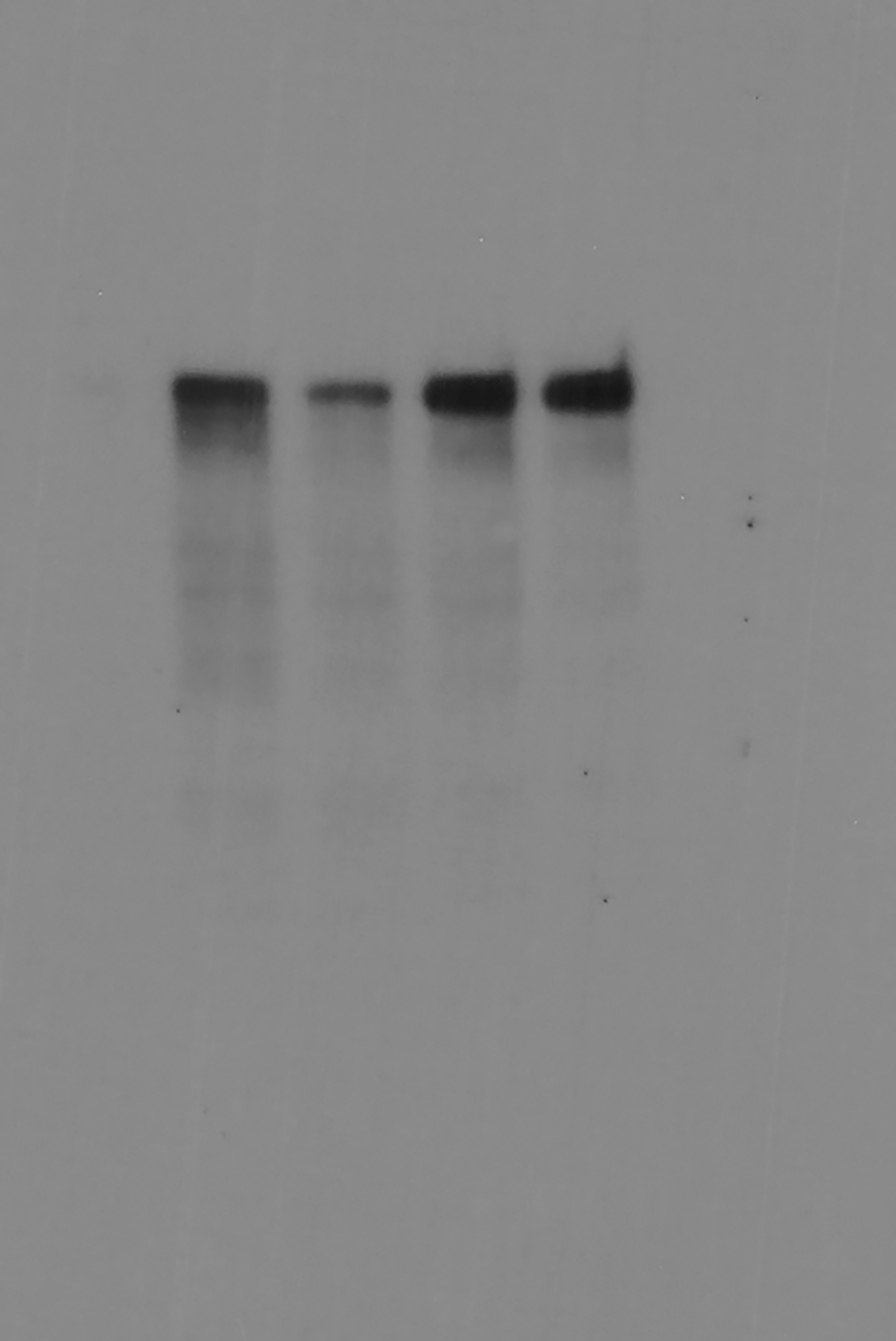

Supplement: Figure 4—source data 2. [file elife-102027-fig4-data2.zip › Figure 4-source data 2/1_Tbx3_2023_05_03_19_34_12-2.jpg]

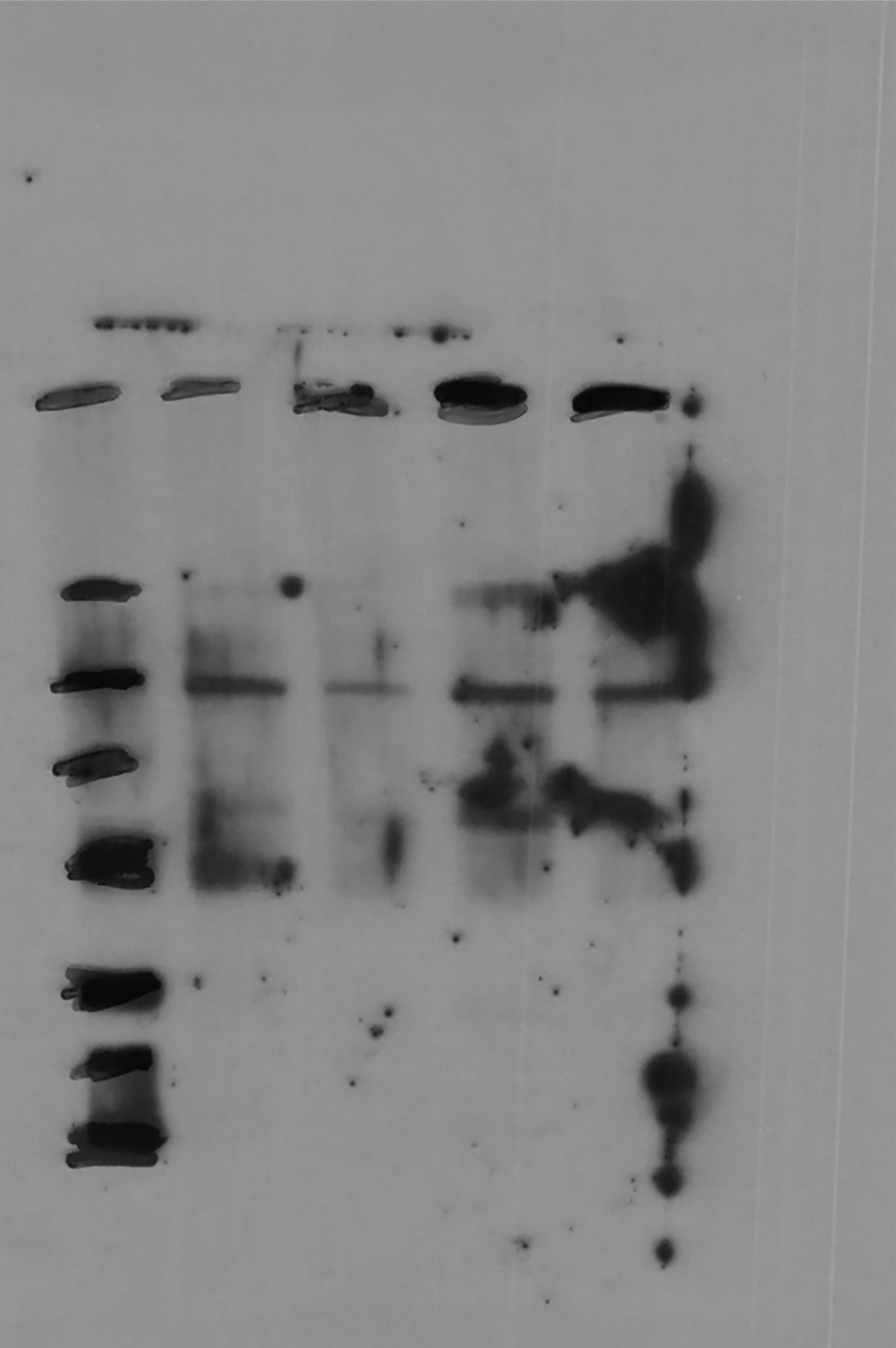

Supplement: Figure 4—source data 2. [file elife-102027-fig4-data2.zip › Figure 4-source data 2/2_HCN4_2023_05_03_18_09_58-2.jpg]

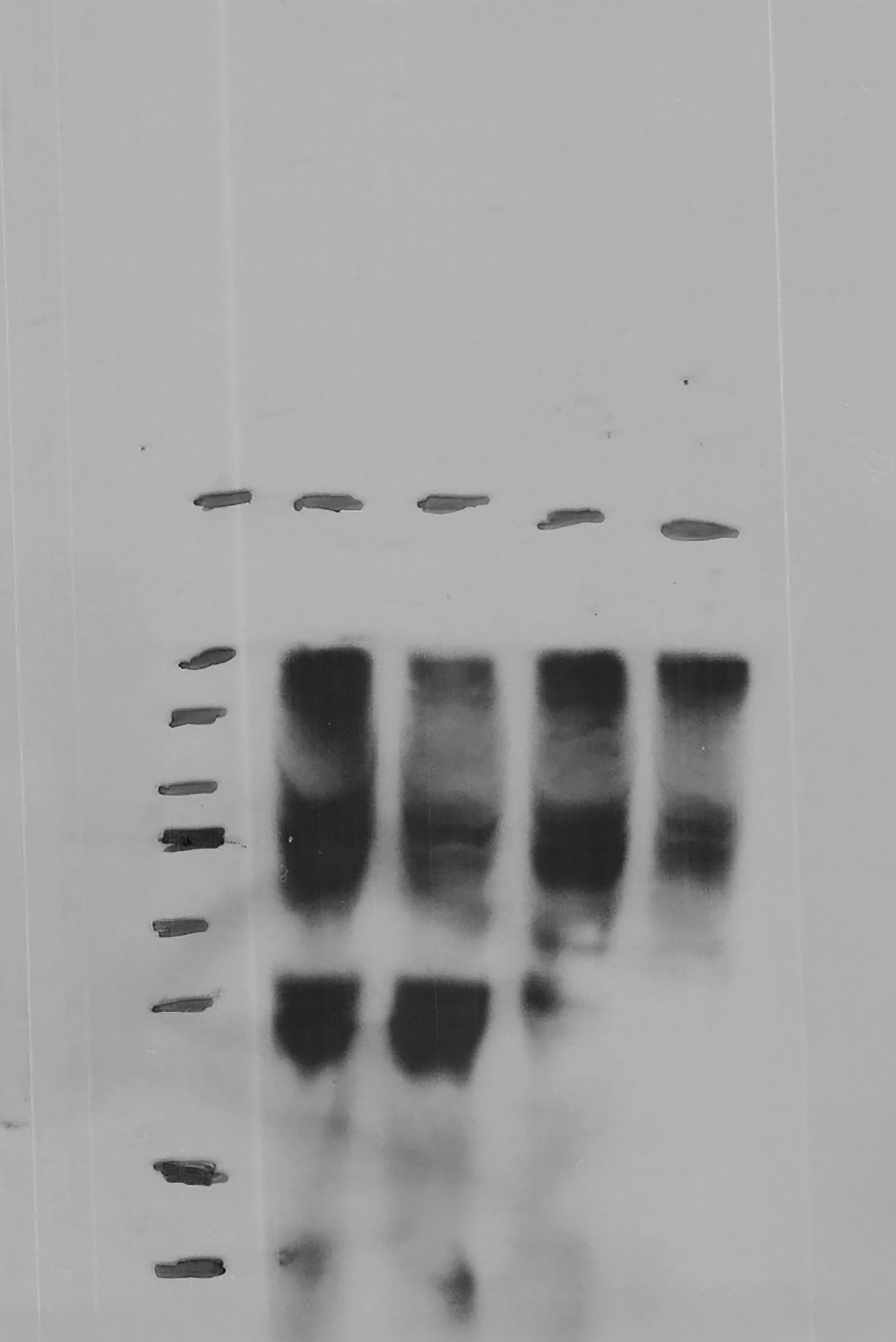

Supplement: Figure 4—source data 2. [file elife-102027-fig4-data2.zip › Figure 4-source data 2/3_Cacna1D_2023_05_03_17_42_13-2.jpg]

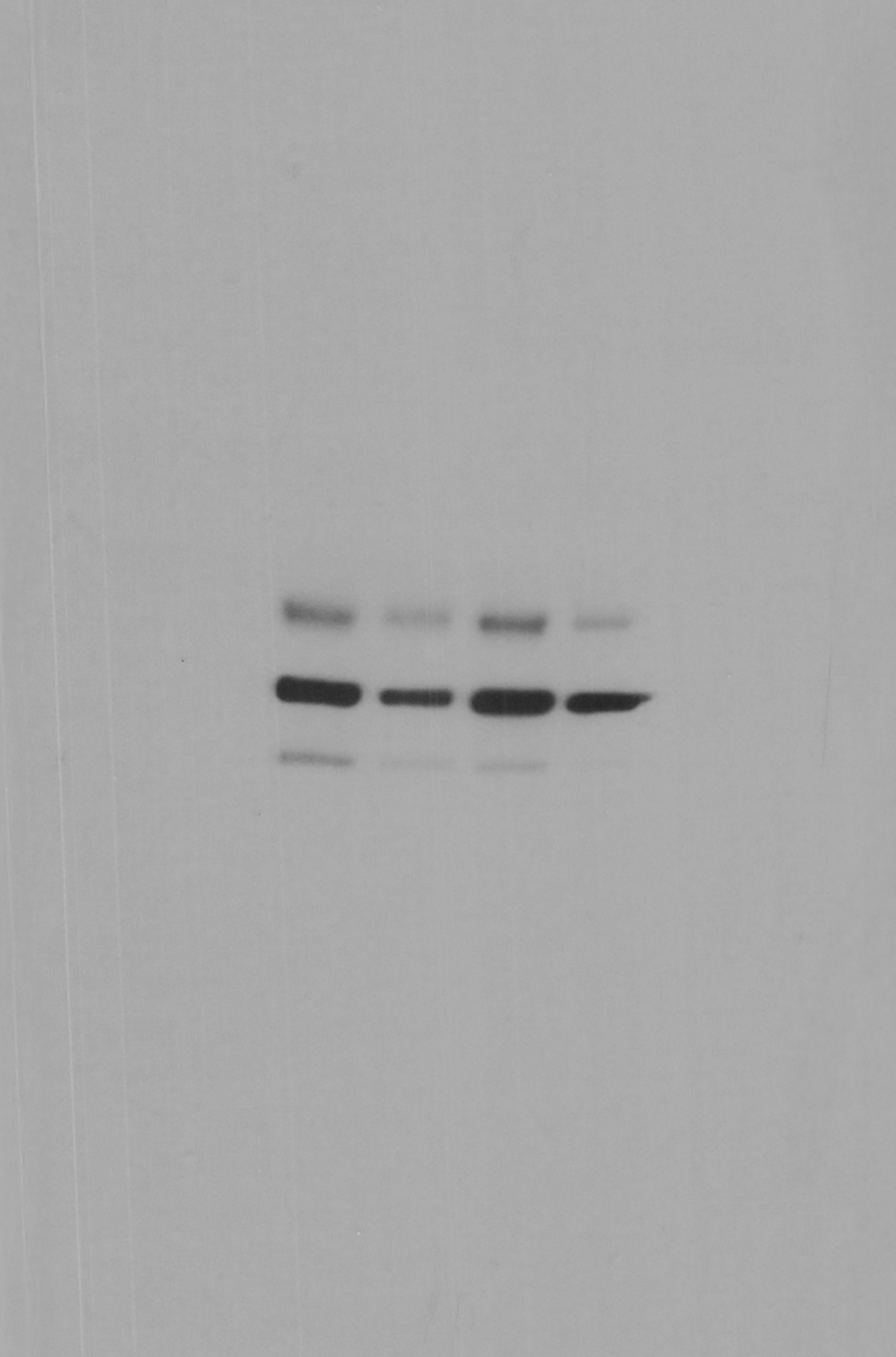

Supplement: Figure 4—source data 2. [file elife-102027-fig4-data2.zip › Figure 4-source data 2/4_GJC1_2023_05_03_20_22_22.jpg]

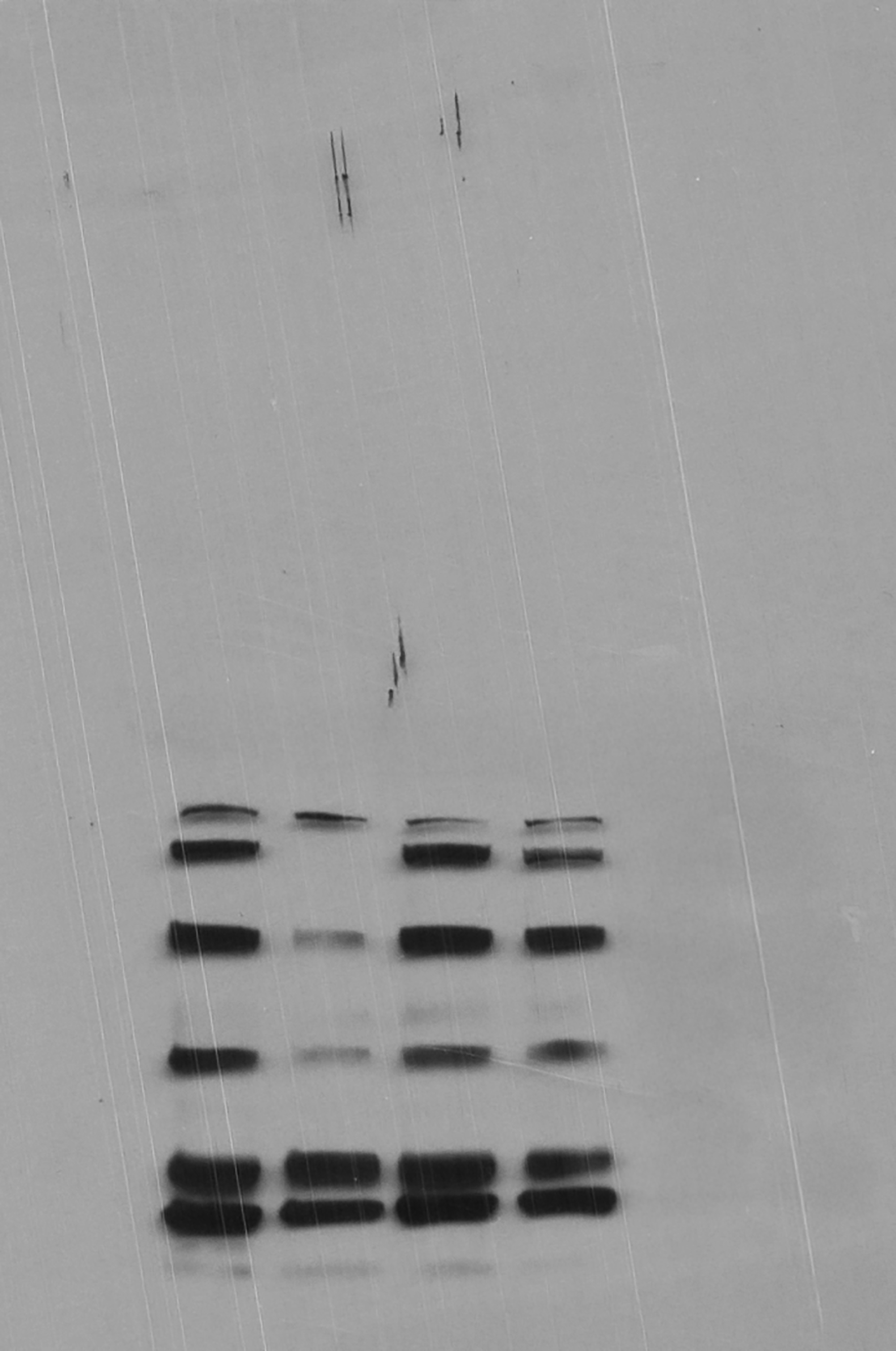

Supplement: Figure 4—source data 2. [file elife-102027-fig4-data2.zip › Figure 4-source data 2/5_Tbx5_2023_05_03_18_36_10-1_2.jpg]

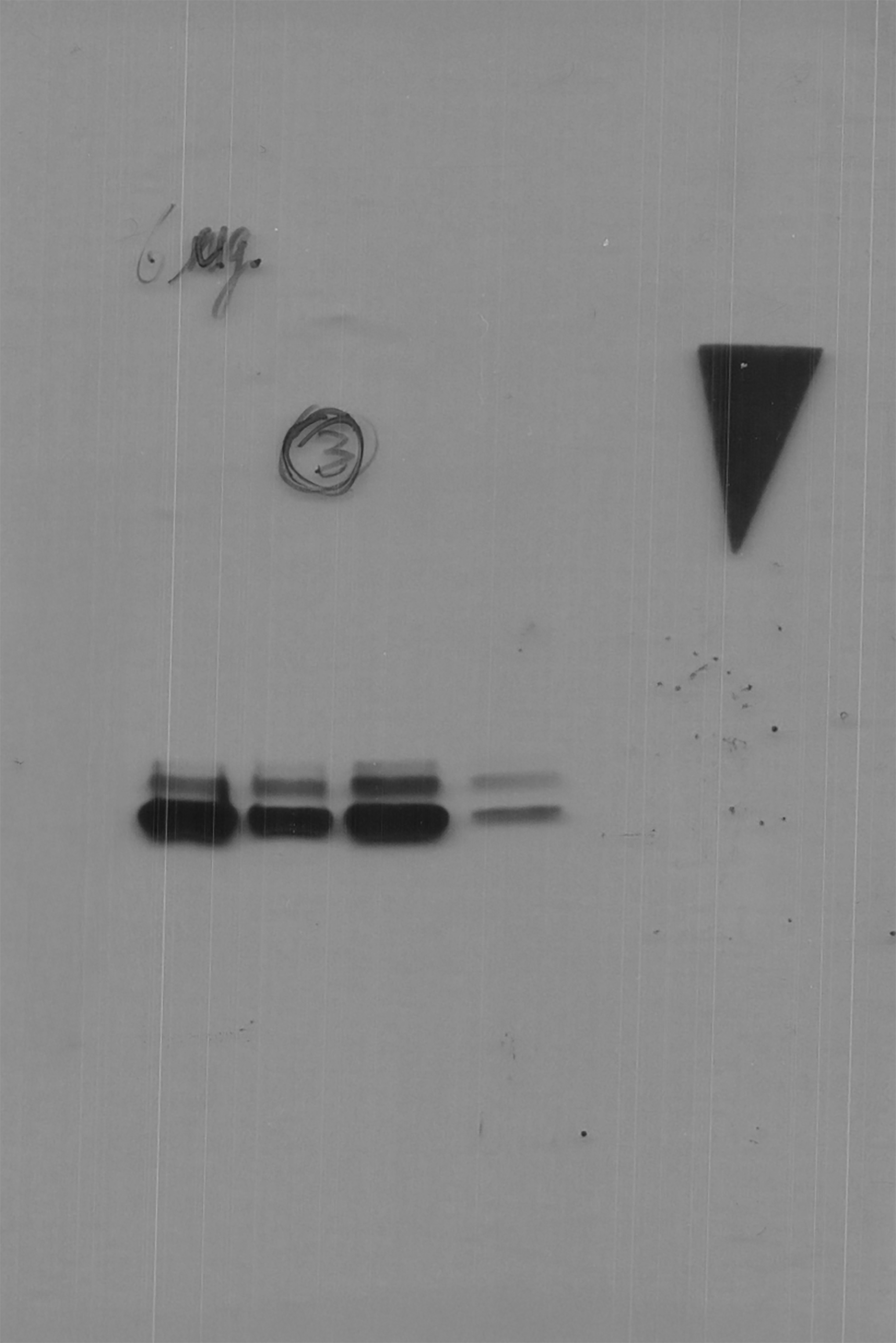

Supplement: Figure 4—source data 2. [file elife-102027-fig4-data2.zip › Figure 4-source data 2/6_GJA5_2023_05_03_07_06_44-1_2.jpg]

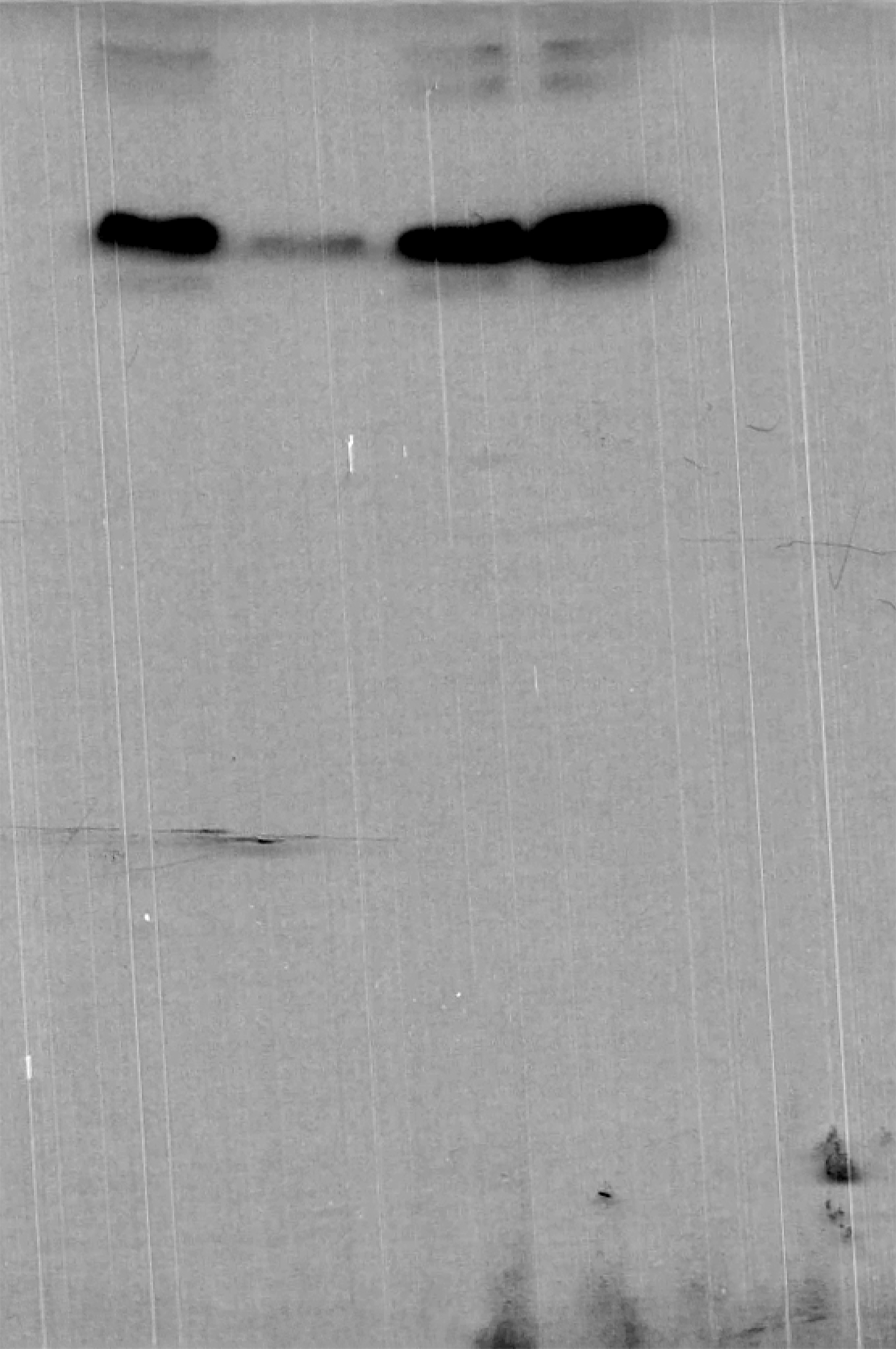

Supplement: Figure 4—source data 2. [file elife-102027-fig4-data2.zip › Figure 4-source data 2/7_Scn5A_2023_05_03_07_11_55-1_2.jpg]

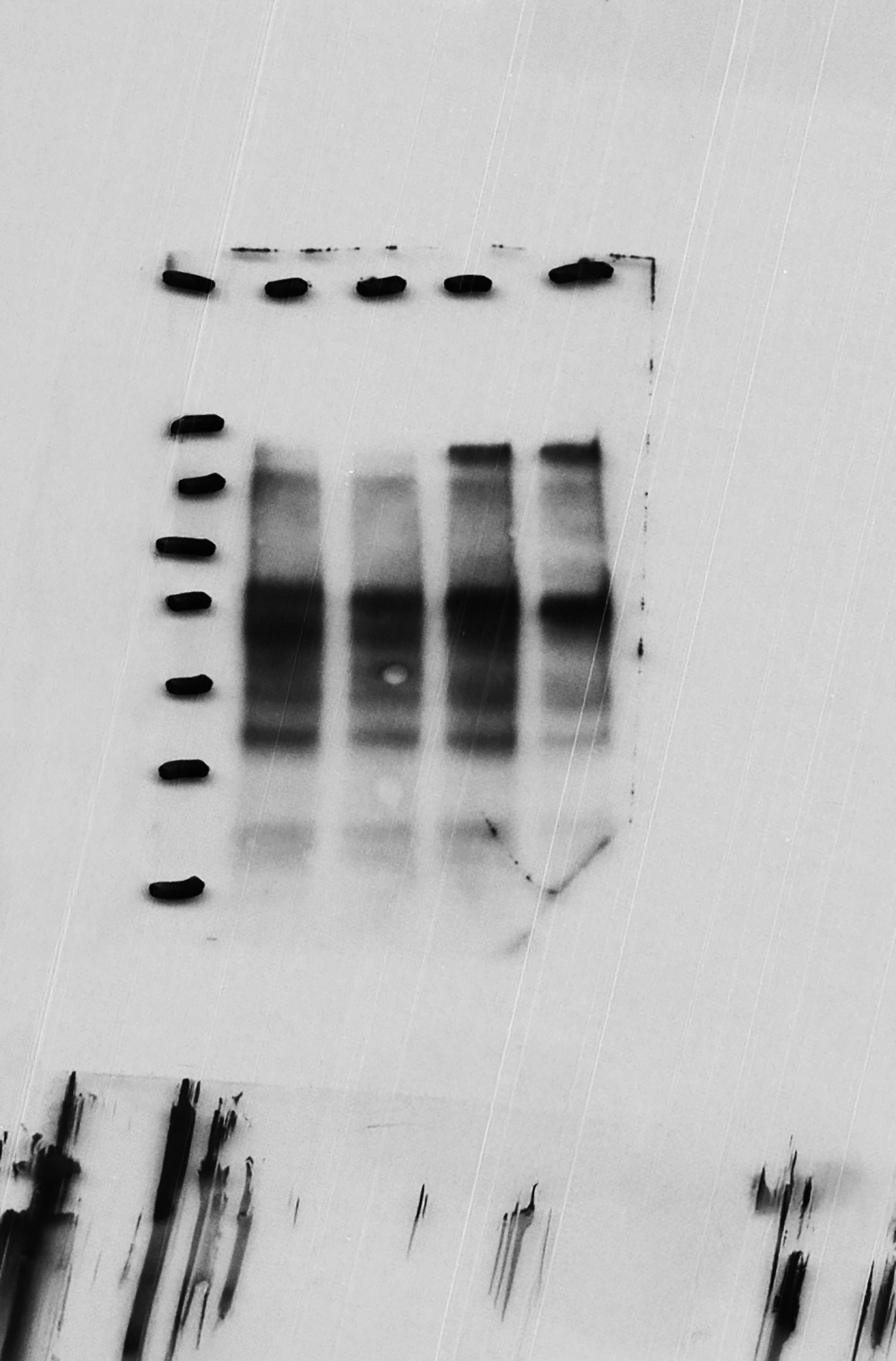

Supplement: Figure 4—source data 2. [file elife-102027-fig4-data2.zip › Figure 4-source data 2/8_Kcnk3_2023_05_03_22_59_53-2.jpg]

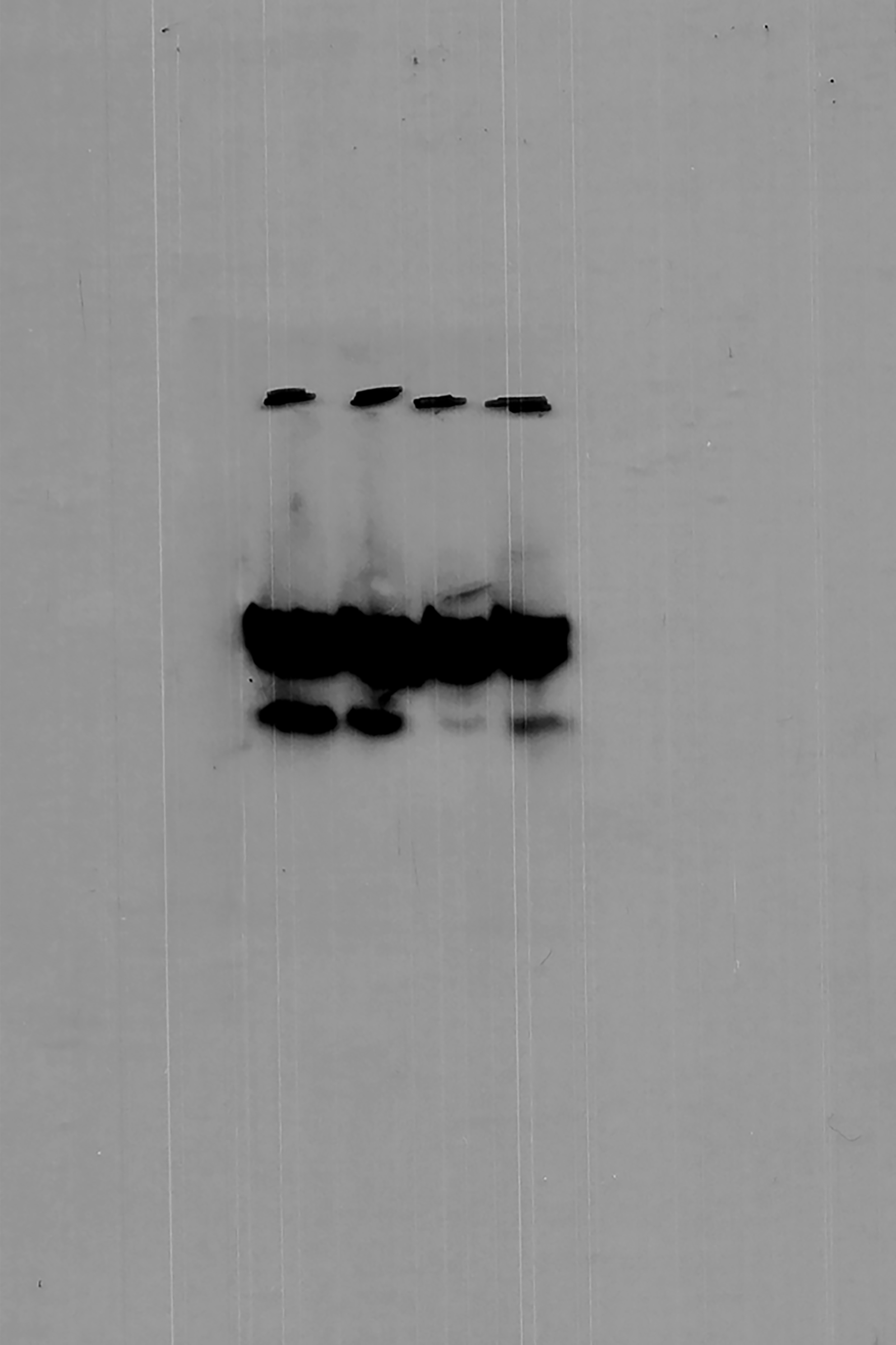

Supplement: Figure 4—source data 2. [file elife-102027-fig4-data2.zip › Figure 4-source data 2/9_GJA1_2023_05_03_20_00_35-1_3.jpg]
