# Supplementary material for: Coordinated Tbx3/Tbx5 transcriptional control of the adult ventricular conduction system
Source: eLife. 2025 Jul 24;13:RP102027. doi: 10.7554/eLife.102027 (PMC12289313; doi:10.7554/eLife.102027)

F1: Genotyping for *Tbx5* locus

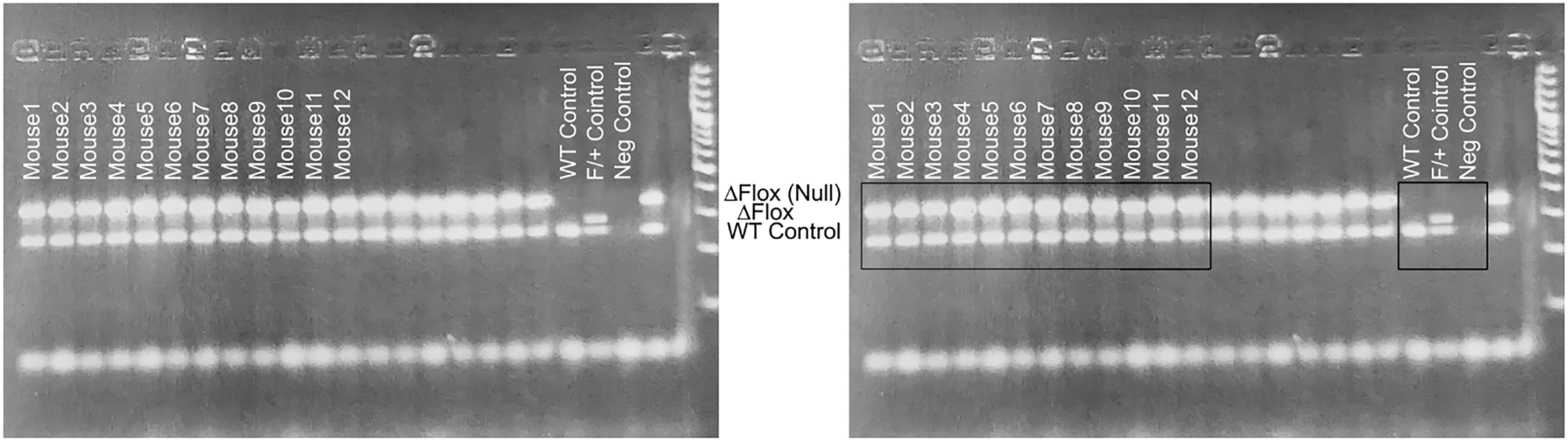

F1: Genotyping for *Cre* locus

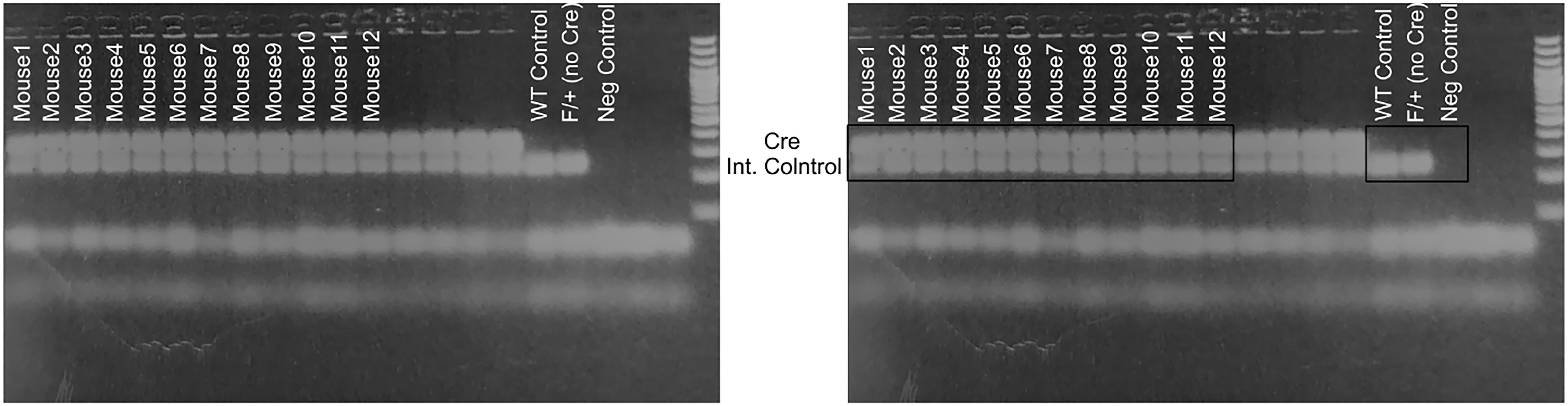

F3: Genotyping for *Tbx5* locus

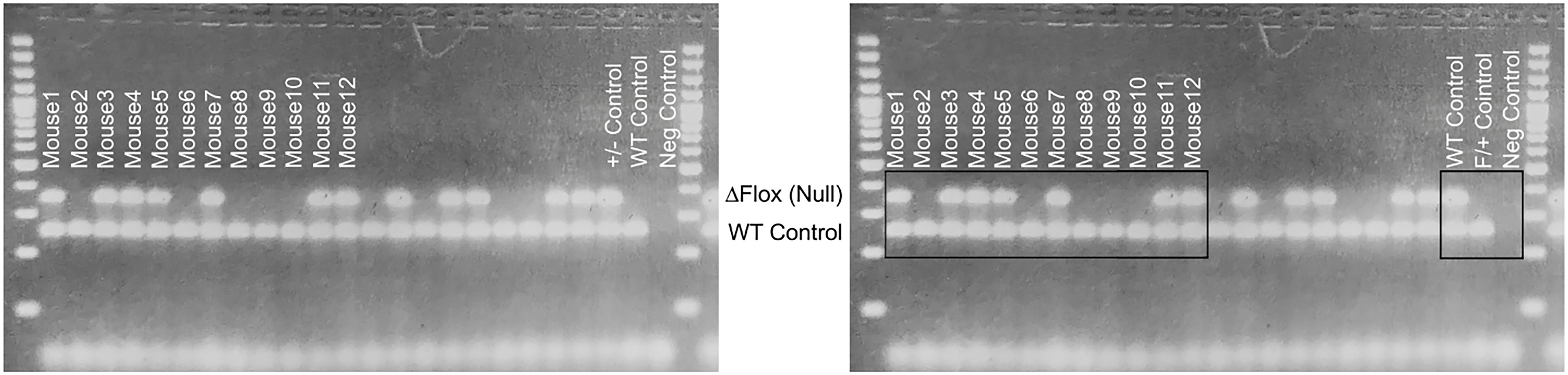

Supplement: Figure 1—figure supplement 2—source data 1. — The black rectangle indicates the region included in the final figure panel. [file elife-102027-fig1-figsupp2-data1.pdf]
